# Molecular Phylogenies indicate a Paleo-Tibetan Origin of Himalayan Lazy Toads (*Scutiger*)

**DOI:** 10.1038/s41598-017-03395-4

**Published:** 2017-06-12

**Authors:** Sylvia Hofmann, Matthias Stöck, Yuchi Zheng, Francesco G. Ficetola, Jia-Tang Li, Ulrich Scheidt, Joachim Schmidt

**Affiliations:** 1 Helmholtz-Centre for Environmental Research, Department of Conservation Biology, Permoserstrasse 15, D-04318 Leipzig, Germany; 2 Leibniz-Institute of Freshwater Ecology and Inland Fisheries, Müggelseedamm 301, D-12587 Berlin, Germany; 3 Chinese Academy of Sciences, Chengdu Institute of Biology, Department of Herpetology, Chengdu, 610041 China; 4 University of Grenoble Alpes, Laboratoire d’Ecologie Alpine, F-38000 Grenoble, France; 5 Natural History Museum Erfurt, Department of Herpetology and Mammalogy, Grosse Arche 14, D-99084 Erfurt, Germany; 6 Philipps Universität Marburg, Department of Ecology, Karl-von-Frisch Strasse 10, D-35032 Marburg, Germany; 7 University of Rostock, Institute of Biosciences, General and Systematic Zoology, Universitätsplatz 2, D-18055 Rostock, Germany

## Abstract

The Himalaya presents an outstanding geologically active orogen and biodiversity hotspot. However, our understanding of the historical biogeography of its fauna is far from comprehensive. Many taxa are commonly assumed to have originated from China-Indochina and dispersed westward along the Himalayan chain. Alternatively, the “Tibetan-origin hypothesis” suggests primary diversification of lineages in Paleo-Tibet, and secondary diversification along the slopes of the later uplifted Greater Himalaya. We test these hypotheses in high-mountain megophryid anurans (*Scutiger*). Extensive sampling from High Asia, and analyses of mitochondrial (2839 bp) and nuclear DNA (2208 bp), using Bayesian and Maximum likelihood phylogenetics, suggest that the Himalayan species form a distinct clade, possibly older than those from the eastern Himalaya-Tibet orogen. While immigration from China-Indochina cannot be excluded, our data may indicate that Himalayan *Scutiger* originated to the north of the Himalaya by colonization from Paleo-Tibet and then date back to the Oligocene. High intraspecific diversity of *Scutiger* implies limited migration across mountains and drainages along the Himalaya. While our study strengthens support for a “Tibetan-origin hypothesis”, current sampling (10/22 species; 1 revalidated﻿: *S. occidentalis*) remains insufficient to draw final conclusions on *Scutiger* but urges comparative phylogeographers to test alternative, geologically supported hypotheses for a true future understanding of Himalayan biogeography.

## Introduction

The Himalaya presents one of the most impressive active orogens on earth, stretching as an arc over 3,000 km from Kashmir (N-Pakistan, N-India) in the West, through Nepal and Bhutan to NE-India and China in the East. The formation of the Himalaya was initiated by the collision of the continental plate of India with Eurasia and ultimately led to the uplift of the Tibetan Plateau. It is widely accepted that this complex geological process started around 45 ± 5 Mya, possibly creating a high elevated terrain (“Paleo-Tibet”), comprising today’s southern Tibet, and a staged further uplift around 25–20 Mya^1–4^ (Supplementary Information S1). The Greater Himalaya has probably arisen subsequently to that of southern Tibet^5^, the earliest in the post-Eocene, or even more recently (~20–10 Mya)^6–8^. However, translating uplift scenarios into paleo-elevations has barely been achieved or applied to biogeographical studies^9^ but is of particular interest for a comprehensive understanding of the distributional history of Himalayan biota.

Biogeographically, the southern slope of the Himalaya forms a transition zone between the Palearctic and Indo-Malayan Realm and is considered a hotspot of biodiversity, fostering a tremendous faunal and floral species richness^10, 11^. Substantial topographic variation as well as the complex and dynamic geological and climatic history contribute to the species’ diversity of this mountain belt. Consequently, patterns and evolution of Himalayan biodiversity are subjects of continuing interest and discussion. In particular, our understanding about the origin and historic biogeography of the terrestrial faunas inhabiting the southern slope of the Greater Himalaya is far from being conclusive. Most phylogeographic studies focussing on these topics, have been conducted in relatively young taxonomic groups and/or groups with high dispersal abilities, such as birds, butterflies, and plants (e.g. refs. 12–16). On the one hand, for the majority of those organisms, origins via long-distance dispersal from the mountains of China-Indochina along the Himalayan chain have been reported, associated with very little speciation *in situ* ^12–15, 17–20^. On the other hand, for several Palearctic faunal elements, influx from the West along a climatically temperate corridor, enabling dispersal from Central Asia and the Pamiro-Alai region into the Himalaya, has been shown^21, 22^. Hitherto, the Himalayan wildlife is considered predominantly as an “immigration fauna”, comprising elements from adjacent faunal realms^21^.

In contrast, a large number of mainly morphological studies, particularly on terrestrial invertebrates, have pointed to the importance of the Greater Himalaya as a centre of *in situ* speciation (summary^21, 23^). In addition, a recent phylogeographic study, based on forest-dwelling, wingless ground beetles, has suggested an alternative hypothesis of the faunal history, namely a south-(Paleo-)Tibetan origin of some Himalayan groups (“Tibetan-origin hypothesis”), suggesting primary diversification of lineages in Paleo-Tibet, and secondary diversification along the slopes of the later uplifted Greater Himalaya. This model is well in line with geological data^24–26^, and also explains apparently paradoxical phylogeographic patterns^27^. In more detail, this hypothesis proposes that recent Himalayan lineages may have evolved from ancestral ones that inhabited the south of the Himalaya-Tibet orogen during its early uplift, when this component of the modern mountain system was still geographically separated from other mountainous regions^27, 28^. If so, adaptation to high altitudes and primary diversification of local species groups would have happened or at least been initiated in the high mountains of South (Paleo-)Tibet, potentially long before the final uplift of the Greater Himalaya. Colonisation of the latter would have taken place in the course of its growth by ancestral species, originating in the immediately adjacent mountains to its north. Due to orogenesis with progressive uplift and aridification of Tibet as a consequence to its location in the monsoonal rain shadow of the growing Greater Himalaya, the primary distribution ranges of many ancestral lineages at the southern edge of (what is now) Tibet have been lost, leading there to faunal extinction or turnover. Such a scenario can best be tested by phylogeographic analyses involving species groups with low dispersal capacity. Nevertheless, based on his comprehensive studies of the High Asian avifauna, Weigold proposed a similar scenario in the middle of the last century^29, 30^.

**Figure 1:**
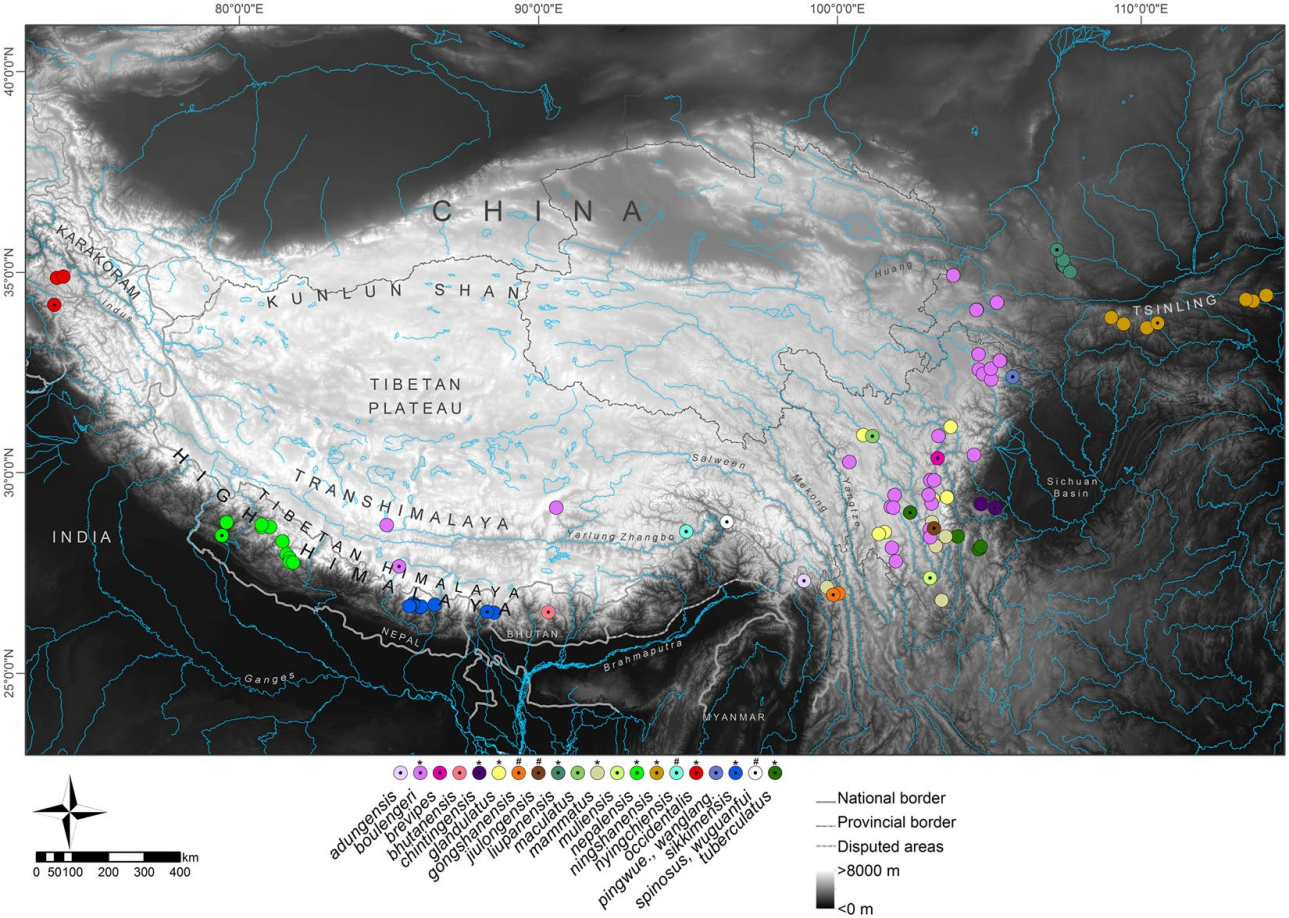
Map of High Asia. Shown are main parts of the Himalayan Tibetan mountain system and known records of *Scutiger* species. Only genetically verified records (coloured circles) and type localities (coloured circles with a dot in the middle) are used, including our samples. For *S*. *bhutanensis*, *S*. *glandulatus* and *S*. *mammatus* georeferencing of the type locality was not possible. For details and further records of *S*. *occidentalis* see Supplementary Information S3. Species included in the concatenated nuclear and/or mitochondrial as well as the *co1*-dataset are marked with an asterisk. A hash key indicates species that were only represented by *co1* data; *pingwue*.  = *pingwuensis*; *wanglang*. = *wanglangensis*. The map was created using ArcMap 10.3.1 (https://www.esri.com/).

In the present paper, we test this Tibetan-origin hypothesis using phylogenetic analyses of mitochondrial and nuclear DNA for anuran amphibians. We focus on the genus *Scutiger* of the Megophryidae, commonly known as “lazy toads”. Megophryidae represent the sister group of Pelobatidae^31, 32^, and form a highly species-diverse family of Oriental anurans with a basal phylogenetic position relative to the Neobatrachia^31, 33–37^. The megophryid lineage has been proposed to originate from the eastern edge of Tibet, in the Hengduan Shan region (*sensu lato*; regions to the east of the Mekong-Salween Divide; Chinese provinces Sichuan, Yunnan, S-Gansu, and SE-Tibet)^38, 39^. All species comprise stream-breeding, forest ground dwellers with toad-like morphology^40^. The genus comprises 21 recognized species, most of which distributed in the Hengduan Shan (Fig. 1); of these 21 taxa, at least five nominal species are only known from their type localities. Several other *Scutiger* species show extremely wide distributions (Fig. 1, Supplementary Information S2 for details).

Due to their relatively low dispersal capacities, adaptation to high elevations and local endemism^41, 42^ *Scutiger* present an ideal anuran model system to test the “Tibetan-origin” (“Himalayan-exile”) hypothesis. If true, we expect that: (i) *Scutiger* from the Greater Himalaya do not show close phylogenetic relationships to lineages occurring at the eastern margin of the Tibetan Plateau, (ii) substructure within Himalayan *Scutiger* will indicate long evolutionary histories of separated lineages, and (iii) geographically separated Himalayan lineages are as old or even older than lineages from the eastern edge of Tibet. Alternatively, assuming a Hengduan Shan origin and long-distance dispersal from the East into the western Himalaya, we except that (i) the phylogenetically oldest lineages to occur in this part of the Himalaya-Tibet orogen, (ii) Himalayan lineages are distinctly younger than those occurring to the east of Tibet, and (iii) lineages from the Western Himalaya are phylogenetically younger than those from the eastern parts of this mountain chain. Here, we strengthen support for a “Tibetan-origin hypothesis” but find that current sampling remains insufficient to draw final conclusions on *Scutiger* and urge comprehensive comparative phylogeographic approaches to decipher Himalayan biogeography in the future.

## Results

### Sequence data

The aligned concatenated sequences (including outgroups) used for phylogenetic inference comprise 10 *Scutiger* and three outgroup species (90 sequences) with 2,839 bp for the mitochondrial and seven *Scutiger* species (55 sequences) with 2,208 bp for the nuclear genes. The mtDNA dataset contained 1,115 variable and 881 parsimony informative characters; the nuDNA contained 121 variable and 88 parsimony informative sites. The mtDNA sequence alignment for divergence time estimations comprised 2,747 bp with 1,378 variable and 1,157 parsimony informative characters.

### Phylogenetic analyses and divergence times

**Figure 2:**
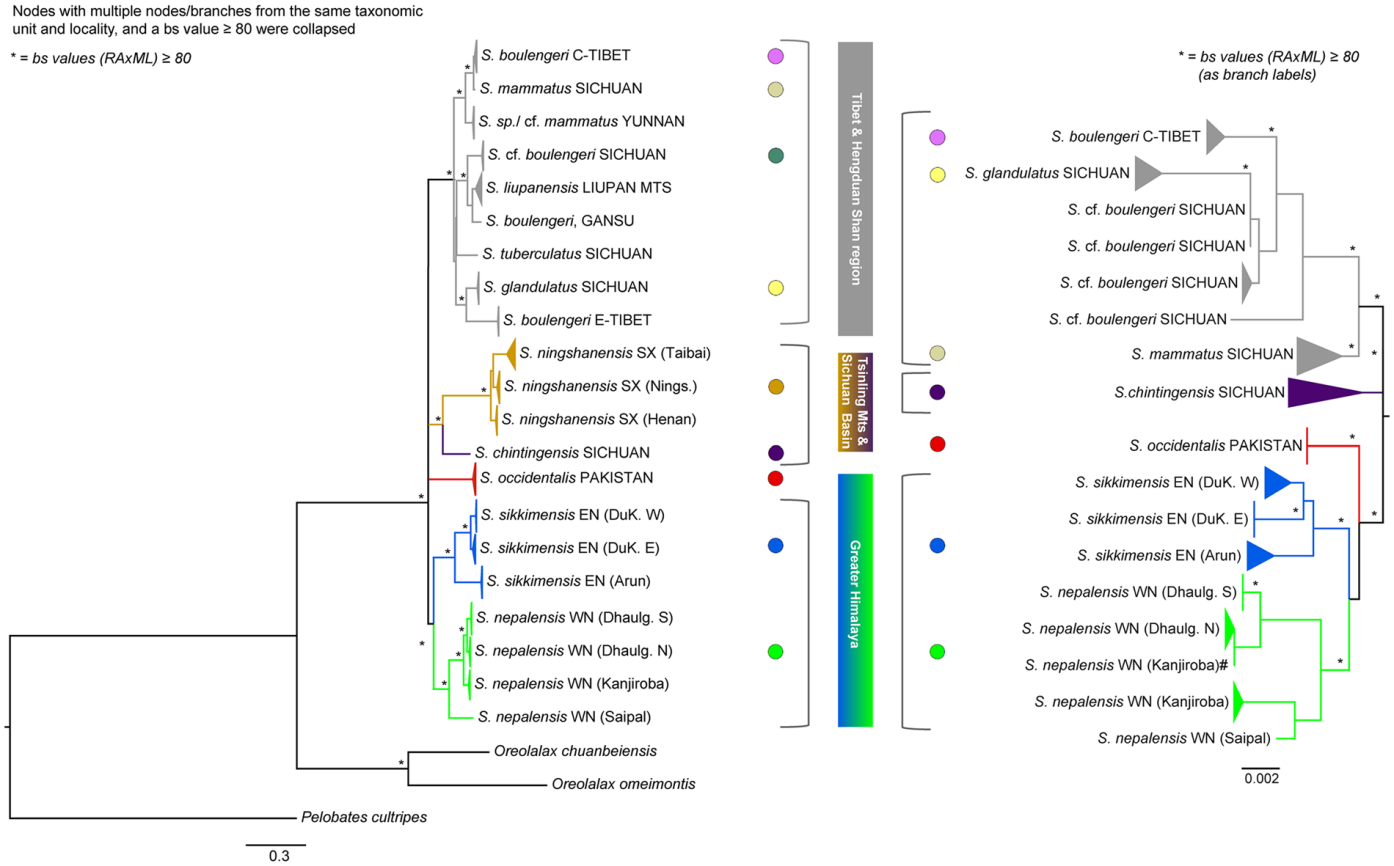
Phylogenetic trees based on mitochondrial and nuclear markers inferred by maximum-likelihood (ML) analyses. Phylograms were obtained with the program RaxML^88^ based on the combined *16 s*, *co1*, *cyt* and *nd4* mtDNA data (left) and the *bfib7*, *ccnb2* and *rag1* nuDNA data set (right). *Scutiger* (*S*.) is followed by the species name and by locality information (Table S5 for sample IDs). An asterisk above branches indicates bootstrap support values ≥80. For clarity, branches representing individuals belonging to the same taxonomic unit were collapsed. Colour codes correspond to those of localities in Fig. 1 and Supplementary Fig. S3. ^#^One sample (A2014-13) from Kanjiroba Himal was placed differently in the mtDNA and nDNA tree (for details text). Inconsistencies occurring between samples in mitochondrial *vs*. nuclear trees are explained by availability of either samples or only sequence information from previous work (for details Table S5). C-Tibet = Central Tibet; Dhaulag. N/S = Dhaulagiri North/South; DuK.E/W = Dudh Koshi River East/West; E-Nepal = East Nepal; E-Tibet = East Tibet; Liupan Mts = Liupan Mountains; Nings. = Ningshan; SX = Shaanxi.

The Bayesian (BI) and maximum likelihood (ML) phylogenetic reconstructions based on the concatenated nuclear or mitochondrial datasets, respectively, were well-resolved and yielded mostly concordant tree topologies with respect to the major clades recovered (Fig. 2, Supplementary Fig. S1). Although some basal nodes and branches differed, and were weakly supported, all analyses supported a (southern) Himalayan clade and another that comprised lineages of the Hengduan region and Tibet (=Tibet-Hengduan Shan clade), except for *S*. *chintingensis* that either clustered separately (nuDNA) or with *S*. *ningshanensis* (mtDNA). In the mtDNA-analyses, the placement of this “Tsinling Mountains & Sichuan Basin” clade as well of *S*. *occidentalis* from Pakistan (so far synonymized with S-Tibetan *S*. *nyingchiensis;* Supplementary Information S3) were poorly supported and remained incongruent in trees resulting from different phylogenetic approaches (Fig. 2, Supplementary Fig. S1). Importantly, all nuclear phylogenies assigned *S*. *occidentalis* consistently with the Himalayan clade, while *S*. *chintingensis* was basally placed, relative to Tibet-Hengduan Shan clade, each with high bootstrap support using ML and BI. Since the root of the nuclear ML tree had to be placed by midpoint rooting (Methods), we tested the resulting topologies of major clades (m1-m3; unconstrained best tree obtained using BEAST [m1], MrBayes [m2], and RAxML [m3.1-m3.6] analyses) in a ML framework based on the mtDNA using the approximately unbiased (AU)^43^ and Shimodaira-Hasegawa (SH)^44^ tests. We included the clade relationships of the nuclear trees as further alternative topology (m4; Supplementary Table S1). We estimated the eight phylogenies as above and the per-site likelihoods in RAxML 8.2.7^45^. The p-values were then obtained using the program CONSEL^46^. We also tested each hypothesis using a Bayes Factor (BF) approach. The marginal likelihoods for the BF calculations were estimated under each model based on both the stepping stone (ss, ref. 47) and path sampling (ps, ref. 48) methods implemented in BEAST using 100 million generations, a chain length of 0.5 million and 50 path steps. Statistical support was then evaluated via 2lnBF using the ps/ss results as per^49^. Although the p-value was the highest for model 3.1 (*S*. *occidentalis* as sister clade to all other *Scutiger* taxa) in both the AU and SH test none of the topologies were rejected, while the BF model selection preferred model m3.3 (*S*. *occidentalis* as the basal sister clade to the Tsinling Mountains-Sichuan Basin clade and the Himalayan clade; Supplementary Fig. S2) based on both the stepping stone and path sampling method (Supplementary Table S1).

Within the Himalayan clade, we recovered additional subclades (Fig. 2, Supplementary Fig. S3, Discussion).

It is noteworthy that several nominal *S*. *boulengeri*, obtained from GenBank resources and during our study, are phylogenetically nested among different lineages within the Tibet-Hengduan Shan clade (Fig. 2). Support values (Bootstrap; BS) for the placement of these *boulengeri*-lineages were partly high (BS ≥ 80), at least in the mtDNA-based ML tree.

Interspecific genetic distances among nominal *Scutiger* species ranged from 5.1% to 13.8% for mitochondrial sequences and from 0.6% to 1.5% for nuclear data (Supplementary Table S2; noteworthy, the mtDNA distances among *S*. sp. and *S*. cf. *mammatus* from Yunnan^50^ and *S*. *mammatus* from Sichuan were at conspecific levels). Between the *S*. *nepalensis* subclades the uncorrected p-distances ranged from 2.2% to 6.6% for mtDNA and from 0.3% to 0.6% for nDNA, while those between subclades of *S*. *sikkimensis* reached 6.5% and 0.4% (data not shown). Within the Tibet-Hengduan Shan clade, the patterns are less clear, but the Central Tibetan lineage (*S*. *boulengeri*) is consistently recovered as distinct subclade in both, the nuclear and the mitochondrial data analyses, with the closest relatives being *S*. *mammatus* (mtDNA) and *S*. *glandulatus*/*S*. *cf*. *boulengeri* from Sichuan (nuDNA).

**Figure 3:**
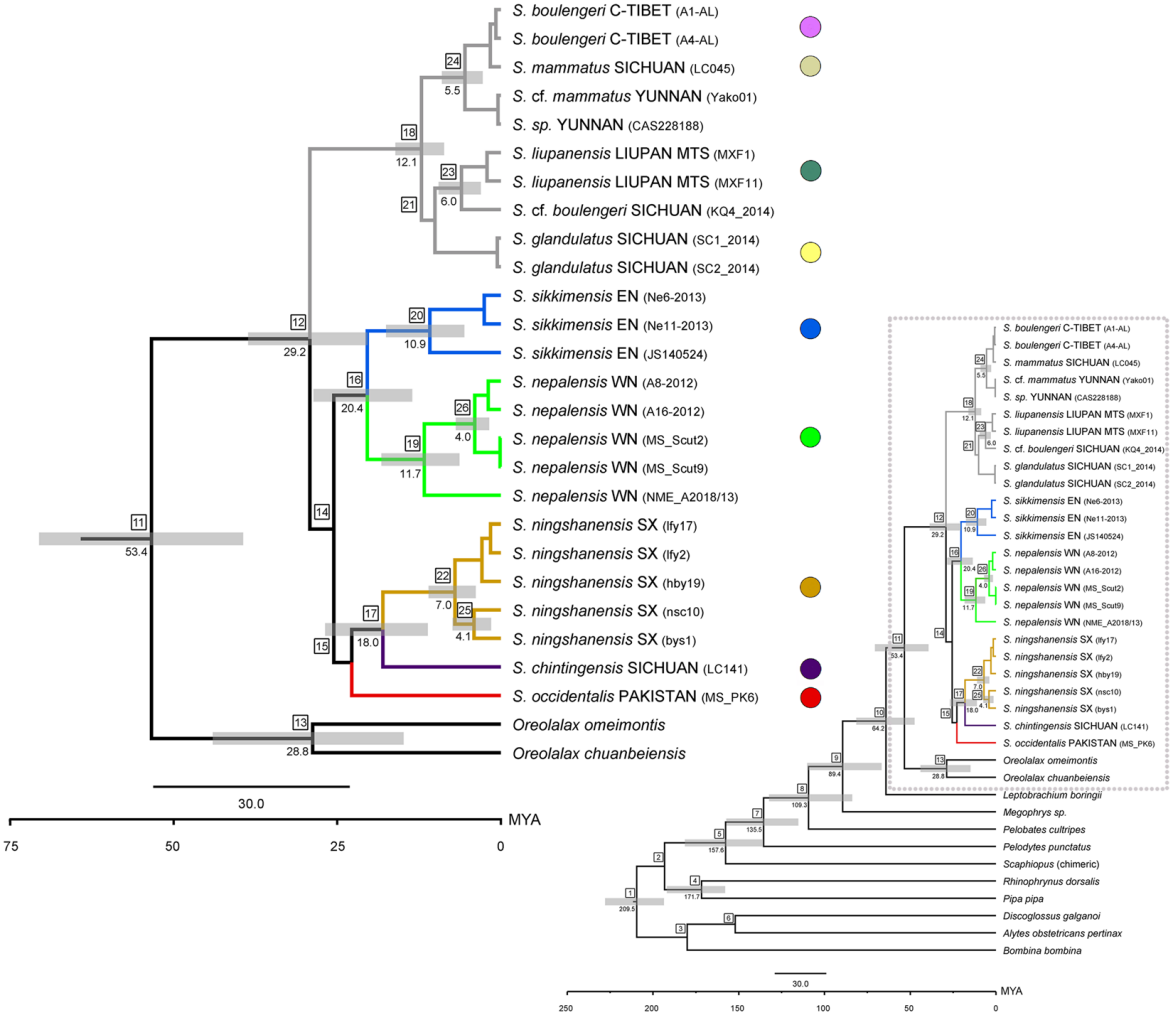
Time-calibrated phylogeny based on the combined *16 s* + *co1* + *cytb* + *nd4* mtDNA data analysis. The divergence times (in Mya) were estimated using fossil calibration points. Dates are only shown for well supported nodes (posterior probability values ≥0.95) and for nodes ≥4 Mya. Node numbers correspond to Supplementary Table S3 that specifies the individual values and 95% highest posterior density intervals (HPDI).

Molecular dating using the expanded taxa set yielded a tree topology and estimates for deep nodes (older than *ca*. 100 million years ago [Mya]) that roughly agreed with other recent studies of anuran divergence times^32, 36, 51, 52^ (Fig. 3, Supplementary Table S3).

Our chronogram places the early diversification of the *Scutiger* to early Eocene (MRCA of *Scutiger* and *Oreolalax* 53 Mya; Fig. 3; Supplementary Table S3). Accordingly, the divergence between the Tibet-Hengduan Shan clade and the remaining *Scutiger* clades was inferred to have happened during the upper Oligocene (29 Mya). Given the uncertain position of *S*. *occidentalis* and the *S*. *ningshanensis*/*S*. *chintingensis* group, the estimated branching of these taxa was not specified. Yet, the W-Himalayan *S*. *occidentalis* clustered basally or at least ancestrally to one of the three distinct clades. The divergence between *S*. *sikkimensis* and *S*. *nepalensis* occurred at *ca*. 20 Mya, while the Tibetan *S*. *boulengeri*-clade and *S*. (cf.) *mammatus*/*sp*. split off within the Tibet-Hengduan Shan clade in mid Miocene (12 Mya). Among *S*. *nepalensis* and *S*. *sikkimensis* further divergence occurred between 12 and 4 Mya resulting in the separation of the Saipal Himal group (12 Mya), the Kanjiroba and Dhaulagiri group (4 Mya), as well as the group in the Arun and the Dudh Koshi catchments (11 Mya), respectively (Fig. 3 and Supplementary Table S3).

### *Co1*-sequence divergences, *Scutiger* species from W-Himalaya (Pakistan)

Uncorrected pairwise genetic distance of the *co1*-gene between specimens of *S*. *nyingchiensis* from Nyingchi, China, and the up to now synonymized *S*. *occidentalis* from Pakistan (Supplementary Information S3, Discussion) ranged from 12.1 to 12.6% (Supplementary Table S4). Similar (interspecific) distances were observed among most of the other *Scutiger* species (up to 16.9%).

In concordance with the results from concatenated mtDNA as well as the nuclear dataset, high intraspecific distances were found between samples of *S*. *nepalensis* from the Saipal Himal and the Dhaulagiri as well as the Kanjiroba Himal (7.2–7.6%). Likewise, remarkably high intraspecific distances were observed within *S*. *sikkimensis*, both, between the groups of the Arun and Dudh Koshi River system (9.0%) and between these two Nepalese groups and samples of *S*. *sikkimensis* from southern Himalaya regions in Yadong, Tibet (12.4–13.1%). Finally, higher intraspecific distances were also found among haplotypes of *S*. *ningshanensis* (3.1–6.3%) and *S*. *boulengeri* (3.8–7.7%).

Based on identical *co1*-sequence data^53^, BI analyses yielded a tree that was largely consistent with these previous results, except for the position of *S*. *ningshanensis* that remained uncertain in our analyses, while it was strongly supported in the original publication (Supplementary Fig. S4). The ML-tree and the two BI-trees of the extended dataset (i.e. including our samples) recovered inconsistent topologies at higher-level nodes, however, with generally weak support for most of these nodes. Yet, the youngest clade, comprising species from Tibet and the Hengduan Shan region east of the Mekong-Salween Divide (*S*. [cf.] *boulengeri*, *S*. *glandulatus*, *S*. *jiulongensis*, *S*. *liupanensis*, *S*. *mammatus*), was consistently recovered, as was the *gongshanensis*-*nyingchiensis*-*spinosus* clade (Supplementary Fig. S5).

## Discussion

### A Paleo-Tibetan origin of Himalayan *Scutiger* species

So far, the recent Himalayan wildlife is considered as an immigration fauna (most frequently as “Sino-Himalayan” fauna), comprising elements from areas surrounding the main mountain arc and lacking further significant evolution^21^. Our study is the first to report on the molecular phylogeny of an amphibian group, spanning the High Himalaya, to test the alternative “Tibetan-origin hypothesis”. The lazy toads of the genus *Scutiger* are notable for their high-altitudinal distribution, their relatively poor dispersal abilities, and, especially, for their complex local and regional diversity^41, 42^. Our main aims are to (i) provide insights into the historical biogeography of the Himalayan clade of *Scutiger*, and (ii) to investigate, whether the intrageneric phylogeny is consistent with a northern or Paleo-Tibetan origin of *Scutiger*, now distributed on the southern slopes of the Greater Himalaya.

First, the phylogenetic analyses of both, the concatenated mitochondrial as well as the nuclear datasets recovered a distinct Himalayan clade that includes *S*. *nepalensis* and *S*. *sikkimensis* from the southern slopes of the main chain. This clade shows no closer relationships to taxa occurring in areas adjacent to the Himalaya. However, the phylogenetic position of *S*. *occidentalis* from Pakistan (so far synonymized with *S*. *nyingchiensis;* Supplementary Information S3) from the most western Himalaya remains uncertain, presumably either due to a gap in taxon sampling and/or because it represents a line that diverged in an early stage of the evolutionary history of *Scutiger*. Secondly, previous studies have argued for an evolutionary origin of *Scutiger* from the Eastern Tibetan Plateau or the Hengduan Mountains^38, 54^. While based on the nuclear tree an eastern ancestry (clade *S*. *chintingensis* from Sichuan) cannot be ruled out, particularly due to the lack of appropriate outgroup taxa, the Hengduan/Tibet group (clades Tibet-Hengduan Shan and Tsinling Mountains-Sichuan Basin) form a sister group to the Himalayan clade (Fig. 2). If, however, *Scutiger* would have originated in Eastern Tibet or adjacent mountains, the species from the Hengduan Shan (and more specifically: species east of the Mekong-Salween Divide) should be placed basally relative to the Himalaya clade and should be older than the latter. Importantly, the most western known *Scutiger* taxon (*S*. *occidentalis*, next paragraph), regardless of its uncertain relative position in the mitochondrial (Fig. 2) and nuclear trees (Supplementary Fig. S1), clusters in most analyses basally to the Himalayan clade. This does not only support the presence of ancestral lineages in the western Himalaya but seems also a strong hint in support for a potential Paleo-Tibetan origin of *Scutiger* rather than their westward immigration. Diversification within the Himalayan clade occurred slightly earlier than in the Tibet-Hengduan Shan and Tsinling Mountains-Sichuan Basin clade (Fig. 3, Supplementary Table S3). Yet, we acknowledge that the current sampling of *Scutiger*, despite representing with 7 (nuDNA) and 10 (mtDNA) out of now 22 nominal species, the largest so far accomplished phylogeny for this genus, remains insufficient to draw final conclusions.

We consider immigration of ancestral *Scutiger* from adjacent lowlands and hills of the Indian subcontinent into the Himalaya also unlikely, as the genus is completely absent on the subcontinent and in the tropics. Despite further caution is required due to large confidence intervals for molecular dating, our divergence time estimates also support the hypothesis that the evolutionary origin of *Scutiger* lies to the north of the High Himalaya, potentially explaining its colonization from southern Paleo-Tibet. Since the Eocene position of “Tibet” was at tropical to subtropical latitudes^1, 28^, cloud forests as suitable habitats of *Scutiger* could have thrived that were geographically separated from similar habitats in southern Central Asia. If so, *Scutiger*-colonization might date back to the lower Oligocen or even Eocene, i.e. evidently prior to the final uplift of the High Himalaya Range^6–8, 16^. Additional support for the existence of uplifted paleo-surfaces in southern Tibet comes from a recent study in the gekkonid genus *Cyrtodactylus*, showing that ancestral lineages of this genus lived in the proto-Himalayan region by early Eocene^55^.

*Scutiger ningshanensis* and *S*. *chintingensis* form a well-supported clade, stretching across the Tsinling Mountains in the province Shaanxi and the margins of the Sichuan Basin (Fig. 1). Their close relationship is in line with morphological data in the species description of *S*. *ningshanensis* ^56^. Early diversification of both taxa dates back the lower Miocene, coinciding roughly with high mountain formation at the southern Eurasian plate^2, 57, 58^, suggesting that this clade also arose north of the High Himalaya (e.g., the northern Paleo-Tibet). Alternatively, it could represent a more ancestral group.

We have found extensive geographically structured relationships among the Himalayan clade, that are linked to the regions east and west of the Kali Gandaki River valley in Nepal (representing *S*. *sikkimensis*, *S*. *nepalensis*), and, based on the nuclear trees, to the most Western Himalaya (*S*. *occidentalis*), indicating an effective physical separation not only between species but also populations. The Nepalese subclades include several distinct lineages that correspond to separated slopes and valleys within the High Himalaya (Fig. 2). For example, in the mtDNA tree, we observed deep branching between lineages on different mountain ranges and drainage systems, namely the Saipal Himal, the Dhaulagiri Himal and Kanjiroba Himal in *S*. *nepalensis* and the catchment of the Dudh Koshi River system and the Arun River system in *S*. *sikkimensis* (Supplementary Fig. S3). The Dhaulagiri lineage is further subdivided into two groups, north and south of the Phagune La Pass, that are linked to different catchment areas. Likewise, the Dudh Koshi lineage splits into a western and an eastern group that are separated by areas of pronounced relief. All of these lineages are corroborated by nuclear sequence data, except for a sample from the Kanjiroba Himal (sample ID A2014-13; see Supplementary Fig. S3), which clustered to the Dhaulagiri group in the nuDNA analysis (Fig. 2).

As we show, *S*. *nepalensis* and *S*. *sikkimensis* diverged *ca*. 20 Mya, matching far-field deformation in Central Asia and exhumation of Greater Himalayan rocks, caused by the India-Asia collision^57^. These orogenetic processes together with the subsequent uplift of the High Himalayan mountain belt (Introduction) could be linked to the rise of the Asian monsoon system and continuous aridization of southern Tibet^8^. Consequently, primary forest-dwelling ancestors in southern Tibet might have successively gone lost by extinction or forced their range shifts along the mountain slopes, paralleling the transverse valleys of the rising Himalaya^27^. Moreover, the high degree of intraspecific genetic diversity, based on mitochondrial and nuclear sequence data, suggests a long-term isolation of these populations, which might have taken place in the respective paleo-ranges of South-Tibet.

The occurrence of *S*. *boulengeri* on the Tibetan Plateau can be explained by re-colonization from the south-eastern margins of the Tibetan Plateau or the Hengduan Shan, respectively, since the closest relatives occurring in these parts of the orogen. Moreover, *S*. *boulengeri* is the only known *Scutiger* taxon which is adapted to the alpine zone, while all other members of this genus are montane taxa, pointing to stepwise high altitude adaption. The relatively recent divergence time between central Tibetan *S*. *boulengeri* and eastern Tibetan *S*. *mammatus* fits the final uplift stage of the Tibetan Plateau (<5 Mya). According to our and previous^59^ analyses, *S*. *boulengeri* appears paraphyletic or even polyphyletic or represents a species complex^59^ with lineages occurring from Central Tibet to the Hengduan Shan (Fig. 1). We encourage further studies to explore potential explanations for this phenomenon.

Although the results from *co1* probably suffered from the limited number of variable sequence positions, a Tibetan origin of the Himalayan *Scutiger* species seems also likely according to these data since *S*. *wuguanfui* is placed basally to all other *Scutiger* in the BI-analysis (Supplementary Figs S4 and S5). *Scutiger wuguanfui* occurs sympatrically with *S*. *spinosus* ^53^ at the Eastern Himalayan Syntaxis (EHS) along the Yarlung Tsangpo River that cuts through the Himalaya prior to its capture by the Brahmaputra (Fig. 1). This southern slope of the Himalaya is of particular interest not only for geoscientists due to large-scale changes in drainage systems prompted by the Indian–Asian collision, but also for biogeographers due to its great biodiversity^60^. The basal position of *S*. *wuguanfui* would support a colonization of the EHS regions by ancestral lineages that originated in Paleo-Tibet and that were forced to follow the Yarlung Tsangpo suture to the east in the course of the rise of the High Himalaya. Geoscientific evidence shows that a major orogen-parallel river system, the Yarlung Tsangpo–Irrawaddy, existed as far back as 40 Mya (and possibly longer)^61^.

Finally, a Tibetan-origin scenario would most parsimoniously explain the clustering of *S*. *gongshanensis* with the Himalayan species (*S*. *nyingchiensis*, *S*. *spinosus*). Its distribution in the Gaoligong Shan (SE-Himalaya) can either be interpreted as consequence of the clockwise rotation around the EHS caused by the underthrusting of the Indian Plate beneath the Eurasian one^61–63^, or alternatively as an eastward migration. The Mekong–Salween Divide appears to act as a topological barrier for *Scutiger*, similarly for several plant species^20, 64^. The *co1*-analysis (Supplementary Figs S4 and S5), however, generally suffered from the single-locus approach and limited sequence length from genetic databases, and thus yield phylogenetic results with limited accuracy^65–67^.

In conclusion, while our data provide indications in support of the “Tibetan-origin hypothesis” for *Scutiger*, they are not entirely in contrast to an immigration scenario from the east. However, our study highlights the importance of considering alternative and overlooked scenarios in biogeographic analyses that are consistent with geological models. A denser sampling in additional mountain systems of the Great Himalaya and, especially, at upper parts of Himalayan transverse valleys with altitudes well above the tropical zone (>2000 m a.s.l.) will be important in the future to bolster our scenario of diversification of Himalayan lazy toads.

### *Scutiger occidentalis* – a valid West-Himalayan species

As shown above, an important result of the present work is the finding of a probably basal position of the W-Himalayan *Scutiger*. The respective samples were obtained during fieldwork on the Deosai Plateau (W-Kashmir, Pakistan) in 2006 and 2008. Two of us (Ficetola *et* Stöck) independently discovered specimens of the genus *Scutiger*, with the 2006 samples initially assigned to *S*. cf. *nyingchiensis* by Ficetola^68^. The Deosai Plateau is only about 70 km straight-line distance from the type locality of *S*. *occidentalis*, described based on 89 specimens from an altitude of 2,920–2,940 m, close to the village Shukdhari, Sonamarg, Jammu & Kashmir^69^. *Scutiger occidentalis* has been considered as a junior synonym of *S*. *nyingchiensis* ^70^, described from the Nyingchi, more than 2000 km from Deosai along the main chain of the Himalaya in south-eastern Tibet^71^. This wide distributional gap between *S*. *nyingchiensis* and the so far synonymized lineage from Deosai provoked doubts in the synonymy of the Kashmir taxon with *S*. *nyingchiensis*. Indeed, we here demonstrate substantial genetic distances of the *co1*-haplotype (~13%) to *S*. *nyingchiensis* from Nyingchi, China^53^, implying that *S. occidentalis* clearly represents a different species. This raises the number of recognized *Scutiger* species to 22. Given the relatively short geographic distance and connection by the same drainage (Supplementary Information S3) between our sampling sites on the Deosai Plateau and the type locality of *S*. *occidentalis* it is highly probable that the specimens we sampled on Deosai can be assigned to this taxon.

## Materials and Methods

### Ethics statement

Samples were collected in accordance with regulations for the protection of terrestrial wild animals. Our study was approved by the relevant Institutional Animal Care and Use Committee (IACUC), namely by the Ethics Committee of the Chengdu Institute of Biology, Chinese Academy of Sciences, China. Sampling in Pakistan was performed under the permit of the Government of Pakistan, Northern Areas Secretariat (Forest Department; NO.F&A-55/F/2006). Samples from Nepal were obtained under the permits of the Nepal expeditions of the Natural History Museum of Erfurt, Germany^72, 73^.

### Sampling and data acquisition

A total of 63 individual *Scutiger* samples (buccal swabs from adults and subadults; muscle or toe from road kills and scientific vouchers; tail tips from tadpoles) came from scientific collections (Chinese Academy of Science, CAS; Natural Museum of Erfurt, NME) or were collected during field work (2012–2015; Supplementary Table S5 and Fig. S3). These included 31 samples from Nepal, nine from the central part of the Tibetan Plateau, five from Kashmir (NW-Pakistan) and 18 samples from the Sichuan Province, China. Species were identified based on morphological characteristics as described^74–77^. Sampling was conducted taking GPS coordinates and elevation records *in situ* (reference system WGS 84). All samples were stored at −20 °C and, except the swabs, in 96% ethanol.

### DNA extraction, amplification, sequencing and alignment

We extracted total genomic DNA from tissues preserved in ethanol using the Qiagen DNeasy kit (Qiagen Inc.) following the manufacturer’s protocol. Swabs were extracted with the same kit or the PG-AC4 Performagene^TM^ reagent package. We amplified partial sequences of the following four mitochondrial and three nuclear loci via the polymerase chain reaction (PCR): *16 S* rRNA (545 bp), *cytochrome oxidase subunit 1* (*co1*, 668 bp), *cytochrome b* (*cytb*, 985 bp), and *NADH dehydrogenase subunit 4* (*nd4*, 641), as well as *beta-fibrinogen intron 7* (*bfib7*, 505 bp), *cyclin B2 gene intron 3* (*ccnb2*, 775 bp), and *recombination activating protein 1 gene* (*rag1*, 957 bp). Primers and conditions for PCR amplification for five of the seven markers were obtained from the literature (Supplementary Table S6). Novel primers were designed for *nd4* and *rag-1* using Primer3 v.4.0.0^78, 79^; see Supplementary Table S6 for a list of primers used, annealing temperature and sources. Amplicons were purified using a QIAquick PCR Purification Kit (Qiagen, Germany) and sequenced in both directions with the same primers. For *rag1* we used additional internal primers for sequencing (see Supplementary Table S6). We identified heterozygotes in electropherograms of the nuclear loci based on secondary peak calling using Geneious 9.0.5 (http://www.geneious.com ^80^) and the Heterozygote plugin. No evidence, such as premature stop codons, of pseudogenes was detected in the mtDNA data. All newly found haplotypes were deposited in GenBank (accession numbers: Supplementary Table S5).

We aligned new sequences of each gene partition along with available *Scutiger* and appropriate outgroup sequences retrieved from GenBank (see Supplementary Table S5) using Muscle 3.8.31 as implemented in Mega 6.0^81, 82^. For the nuDNA dataset, no appropriate outgroup taxa were available (given the long divergences, intronic noncoding sequences would not allow for unambiguous alignments). All gene fragments were translated into amino acids; no frameshift mutations or premature stop codons were observed. One ambiguous alignment site was excluded (29 bp of a CA repeat, from pos. 269 in *ccnb2*); otherwise gaps were treated as missing data in all subsequent analyses. Nuclear alleles could not be phased because most populations were represented by only a few or single individuals which did not allow a robust statistical inference of haplotypes. Therefore, all polymorphic sites were encoded with the appropriate IUPAC ambiguity code.

### Phylogenetic reconstruction

Prior to phylogenetic reconstruction, we analysed each aligned data set for saturation by plotting the pairwise number of observed transitions and transversions versus the genetic distance. In addition, substitution saturation was evaluated with Xia’s test as implemented in the program DAMBE^83, 84^. For protein-coding loci, codon positions 1 and 2 were analysed together while codon position 3 was treated separately. None of the data sets showed substantial sequence saturation, as indicated by Xia’s test, and a nearly linear relationship of transitions and transversions. Although, third mitochondrial codon positions generally tend to be partially saturated^85^, such partial saturation does not necessarily imply lack of phylogenetic signal. Implementation of partitioning over codon positions and relaxed-clock models has been shown to improve molecular phylogenetic and dating analyses in such circumstances^86, 87^ (see further below). The three mitochondrial sequences were concatenated for subsequent analyses and separately, the three nuclear gene fragments. Sequence duplicates were removed using mothur v. 1.35.1 (http://www.mothur.org/).

Phylogenetic relationships were calculated separately from mitochondrial and nuclear datasets based on Maximum Likelihood (ML) and Bayesian Inference (BI) methods, using RAxML 8.0.0^88^ as well as BEAST 1.8.3^89, 90^ with input files created in BEAUTi 1.8.3 and MrBayes 3.2.6^91^. All phylogenetic analyses were run on the CIPRES Science Gateway^92^.

We created partitions for each gene fragment; protein-coding genes were partitioned according to codon position. To optimize partitions and substitution models, this *a priori* selected scheme was used as input for PartitionFinder 1.1.1^93^. As input settings we selected linked branch lengths, the Bayesian Information Criterion (BIC) in a greedy search algorithm and the BEAST, MrBayes and RAxML option^94^, respectively. BI analyses were then run using models and partitions as selected by PartitionFinder (Table 1). In BEAST, substitution schemes were unlinked between partitions. Prior to analyses, likelihood ratio tests (LRT) were performed to determine whether each gene’s evolutionary pattern conforms to expectations of a global molecular clock. Likelihood scores were calculated without and with enforcing a molecular clock model in the program PAUP*v4.0b10^95^, and significance of the LRT was evaluated by a Chi-square test. Because the strict molecular clock model was rejected for the data sets, we set an uncorrelated log-normal relaxed clock in BEAST. We ran two chains for 20 million generations, with a sampling interval of 2000. Convergence of modelled parameters and potential autocorrelations (effective sampling size for all parameters >200) was confirmed using the software Tracer v.1.7.2^96^. A maximum credibility clade tree was generated using TreeAnnotator v.2.3.2^89^ with a burn-in of the first 25% of the sampled trees, a posterior probability threshold of 0.5 and median node heights. In MrBayes v.3.2.6^91^ the BI-tree was inferred by running 10 million generations with four chains, starting with a random tree and sampling trees every 1000 generations, until reaching an average standard deviation of split frequencies of <0.01.

**Table 1:** Partition schemes and substitution models used for the divergence time analyses.

Analyses	Data set	Partitions	Model
Phylogeny			
BEAST	mtDNA (2839 bp)	p1: 16 s, cytb_cd1, nd4_cd1	HKY + I + G
		p2: co1_cd1	TrNef + I + G
		p3: co1_cd2, cytb_cd2, nd4_cd2	HKY + I
		p4: co1_cd3, cytb_cd3, nd4_cd3	TrN + I + G
	nuDNA (2208 bp)	p1: bfib7, rag1_cd2, rag1_cd3	HKY + I
		p2: ccnb2	HKY + I
		p3: rag1_cd1	HKY
MrBayes	mtDNA (2839 bp)	p1: 16 s, cytb_cd1, nd4_cd1	GTR + I + G
		p2: co1_cd1	K80 + I + G
		p3: co1_cd2, cytb_cd2, nd4_cd2	HKY + I
		p4: co1_cd3, cytb_cd3, nd4_cd3	GTR + I + G
RAxML	mtDNA (2839 bp)	p1: 16 s, co1_cd1, cytb_cd1, nd4_cd1	GTR + I + G
		p2: co1_cd2, cytb_cd2, nd4_cd2	GTR + I + G
		p3: co1_cd3, cytb_cd3, nd4_cd3	GTR + I + G
	nuDNA (2208 bp)	no partition	GTR + I + G
Divergence time			
BEAST	mtDNA (2747 bp)	p1: 16 s	HKY + I + G
		p1: co1_cd1	TrNef + G
		p2: co1_cd2	HKY
		p3: co1_cd3, cytb_cd3, nd4_cd3	TrN + G
		p4: cytb_cd1, nd4_cd1	HKY + G
		p5: cytb_cd2, nd4_cd2	TrN + G

As in the BI analysis, we set the model and partitioning scheme selected by PartitionFinder 1.1.1 for the ML-tree inference (Table 1). Nodal support was assessed by the rapid bootstrapping algorithm with 1000 bootstrap replicates and the GTRGAMMA approximation.

For the nuclear data set, we used BEAST that involves a clock model for rooting and RAxML which allows for midpoint rooting approach. The ML-tree was rooted at the branch that best balances the subtree lengths using RAxML 8.2.7^45^; support values are displayed by branch labels instead of node labels. It must be noted that midpoint rooting can be unreliable, especially if evolutionary rates vary across the taxa being considered^97^. Therefore, the root of the resulting ML-tree must be treated with caution.

### Divergence time estimation

Due to the unavailability of appropriate outgroup sequences for the nuclear intronic data, divergence dates were estimated based on the concatenated mitochondrial data set only, using BEAST 1.7.5^89, 90^. In order to place fossil calibration references, we used several additional out-groups: *Alytes obstetricans*, *Bombina bombina*, *Discoglossus galganoi* (Bombinatoroidea); *Megophrys* sp., *Pelobates cultripes*, *Pelodytes punctatus*, *Scaphiopus*, (Pelobatoidea); *Pipa pipa* and *Rhinophrynus dorsalis* (Pipoidea). Sequences of two spadefoot toads (*co1* of *Scaphiopus holbrookii* and *cytb* of *Scaphiopus couchii*) were combined to obtain a single chimeric outgroup taxon for the dating analyses^36^. These sequences were taken from GenBank (http://www.ncbi.nlm.nih.gov/; Supplementary Table S5). To avoid potential problems with model parameter variance across heterogeneous datasets^98, 99^, we inferred divergence times from a reduced dataset of maximally two individuals from each phylogeographic *Scutiger* lineage and the out-groups.

The best-fitting models of sequence evolution were set using PartitionFinder 1.1.1 and unlinked across partitions (Table 1). We imposed the following calibrations to the molecular clock: (1) A minimum age of 166 Mya for the most recent common ancestor (MRCA) of Discoglossoidea and Pipanura (calibration of the root node), based on the fossil *Eodiscoglossus oxoniensis* ^100, 101^. The upper 95% CI value was set to 252 Mya, corresponding to the maximum age of the salientian *Triadobatrachus* ^102^. (2) A minimum age of 148 Mya for the Rhinophrynidae-Pipidae split, based on the fossil *Rhadinosteus parvus* attributed to Kimmeridgian age^103^ and considering the lower range specification by Canatella^104^. (3) A minimum age of 50.3 Mya of the MRCA of Pelobatoidea, based on the fossil *Scaphiopus guthriei* ^105, 106^. (4) A minimum age of 33.9 Mya of the MRCA of Pelodytidae, Pelobatidae and Megophryidae, based on the fossil *Pelodytes* ^107^. (5) A mean age of 198 Mya for the age of Bombinatoroidea and a 95% prior interval ranging from 164–239 Mya. (6) A mean age of 53 Mya for the age of Megophryidae and a 95% prior interval ranging from 37–76 Mya. This prior interval roughly corresponds to the 95% CI of the estimated age in ref. 36. To address the problem that accuracy of divergence time estimation of shallow nodes may suffer from lower precision we implemented a younger, secondary calibration based on *cytb* ^108^. This previous work focused on recent clades where the molecular clock is better approximated^109^, substitution saturation is less likely to be significant^85, 110^, and rate variation should be weaker^111^. Accordingly, we set a 95% prior interval ranging from 6.4–14.4 Mya for the MRCA of *Scutiger mammatus* and *S*. *glandulatus* (lognormal distribution). The mean and standard deviation of calibration points (1)-(5) were defined such that the prior interval roughly corresponded to the average CI of estimated divergence time of previous studies^32, 36, 51^. For nodes with soft maxima specified by fossils we used only the lower average CI-value from these studies. Taxa groups corresponding to calibration points (2), (4) and (6) were constraint to be monophyletic as were *Scutiger* and *Oreolalax*, since their monophyly has been well established^31, 36, 112, 113^. We also constrained the *S*. *chintingensis*/*S*. *ningshanensis* clade as it was consistently recovered by the ML and BI phylogenetic analyses.

In all approaches, we used a birth-death process as model of speciation and a random tree as starting tree. We selected a time to the most recent common ancestor (tmrca) prior and root heigh with a lognormal prior distribution and an offset equal to the minimum age of the oldest fossil. Each run was performed with 200 million generations, sampling 10.000 trees and with a burn-in set to 25% of the samples. Convergence and stationary levels were verified with Tracer v1.7.2. We annotated the tree information with TreeAnnotator v.2.3.1 and visualized it with FigTree v.1.4.2^89^.

### Records and identification of *Scutiger* species from Pakistan

To evaluate whether *S*. *occidentalis* is a valid taxon or a synonym of *S*. *nyingchiensis* we analysed the *co1*-sequence data of our samples from Pakistan along with sequences of *Scutiger* species that have been recently released by NCBI (KU243053-KU243067^53^), among them *S*. *nyingchiensis* from Nyingchi, TAR, China. We also included at least one sequence of all taxa and subclades of our sample from East- and West-Nepal (*S*. *sikkimensis*, *S*. *nepalensis*), Sichuan (*S*. cf. *boulengeri*, *S*. *chintingensis*, *S*. *glandulatus*, *S*. *mammatus*) and the Tibetan Plateau (*S*. *boulengeri*) as well as further sequences (GenBank; Supplementary Table S5). The sequence alignment was trimmed to the shortest sequence (556 bp). Uncorrected pairwise genetic distances were quantified using Mega 6.0^81^.

Phylogenetic estimations were again obtained under a Bayesian framework in MrBayes 3.2.6^91^ and BEAST 1.8.3^90^ as well as via a ML approach in RAxML 8.0.0^88^. We applied mixed models according to a codon-based data partition scheme and substitution parameters as specified using PartitionFinder 1.1.1^93^. Analyses were conducted as described above, except that BEAST runs were performed with 50 million generations. ML analyses were performed under a GTR model with gamma-distributed rate variation across sites, a GTRGAMMA approximation for the bootstrapping phase and 1000 bootstrap replicates.

## Electronic supplementary material

Supplementary Information
