# Supplementary material for: Molecular Phylogenies indicate a Paleo-Tibetan Origin of Himalayan Lazy Toads (Scutiger)
Source: Sci Rep. 2017 Jun 12;7:3308. doi: 10.1038/s41598-017-03395-4 (PMC5468327; doi:10.1038/s41598-017-03395-4)
Supplement: Supplementary file 1 — Supplementary Information [file 41598_2017_3395_MOESM1_ESM.pdf]

Supplementary Information

Molecular Phylogenies indicate a Paleo-Tibetan Origin of Himalayan Lazy Toads (*Scutiger*)

Sylvia Hofmann, Matthias Stöck, Yuchi Zheng, Francesco Gentile Ficetola, Jia-Tang Li,

Ulrich Scheidt, Joachim Schmidt

Supplementary information S1

The collision of the continental plates of India and Eurasia was the major geological process that initiated the formation of the Himalaya and the ultimate uplift of the Tibetan Plateau. Although various lines of geoscientific and biogeographic evidence have suggested different uplifting scenarios for the Himalaya-Tibet orogen, the time of the onset of the orogeny, approximately 45±5 Mya, is widely accepted (for review:[1-5]). However, translating evidence for this uplift into paleo-elevations has barely been achieved and thus could hardly be applied to biogeographical studies[6]. Many studies suggest a highly elevated terrain in the region of today's southern Tibet and a multi-stage collision history, e.g. with a "soft" (micro)continent–continent collision in the early Eocene and a "hard" India–Eurasia collision that happened at ~25–20 Mya or earlier[2,3,7-12]. Accordingly, this terrain was uplifted due to significant pre-collisional crustal thickening and has characterized the physiography of the southern margin of Asia from at least 45 Mya (see[1-3,5,10] and references therein), probably forming an Andean-type volcanic mountain chain[13-15]. With the continued continent-continent (hard)[7] collision, the uplift expanded mainly northwards and eastwards. Regardless, which model of collision is assumed, the topography of the Greater Himalaya has obviously arisen subsequently to that of southern Tibet[16] during the post-Eocene at the earliest, possibly even during a more recent period (~20-10 Mya)[11,17,18]. Although important questions remain regarding the spatio-temporal distribution of regions with certain altitudes in the course of the Himalaya-Tibet orogen evolution, the latter point seems of particular interest for biogeographers, who try to understand the distributional history of Himalayan biota, as addressed in the present paper.

Supplementary information S2

Among lazy toads, *Scutiger boulengeri* (Bedriaga, 1898) has the widest known distribution, spanning along the northern slope of the Himalaya, from northern central Nepal[19] through the southern and eastern parts of the Tibet to the Qinghai, Gansu and Sichuan Provinces in W-China[20]. A detailed account of *S. boulengeri* has recently also provided from the Gurudongmar Lake, likewise located on the northern slope of the Himalaya, close to the Indo-Tibetan border[21]. Several species records, e.g. available from GBIF.org, have to be critically reviewed and potentially revised[22-24]. Given the substantial morphological variation and cryptic genetic diversity within the genus *Scutiger* (e.g., within *S. boulengeri*[25]), a verification of the species identification by detailed morphological and/or

molecular analyses seems mandatory in this group. Therefore, we included in our species map (Fig. 1) only the type localities (as far as geo-referencing was possible) and records that have been genetically verified.

As yet, only a few nominal species have been described from the southern slope of the Himalayan range, namely *S. nepalensis* Dubois, 1974, *S. sikkimensis* (Blyth, 1855) and *S. occidentalis* Dubois, 1978 (up to now considered a junior subjective synonym of *S. nyingchiensis* Fei, 1977, which is only known from counties in the south-east of Tibet and from North India[26,27]). Moreover, *S. spinosus* and *S. wuguanfui* have been recently described and show a distribution at the southern side of the Himalaya[28,29]. For the sake of completeness, *Scutiger bhutanensis* should be considered as Himalayan species, although it is known only from the imprecise type locality, "Bhutan", without additional information[30].

References

1 Lippert, P. C., van Hinsbergen, D. J. J. & Dupont-Nivet, G. Early Cretaceous to present latitude of the central proto-Tibetan Plateau: A paleomagnetic synthesis with implications for Cenozoic tectonics, paleogeography, and climate of Asia. *Geological Society of America, Special Papers* **507** (2014).

2 Gibbons, A. D., Zahirovic, S., Müller, R. D., Whittaker, J. M. & Yatheesh, V. A tectonic model reconciling evidence for the collisions between India, Eurasia and intra-oceanic arcs of the central-eastern Tethys. *Gondwana Research* **28**, 451–492 (2015).

3 Li, Y. *et al.* Propagation of the deformation and growth of the Tibetan–Himalayan orogen: A review. *Earth-Science Reviews* **143**, 36–61 (2015).

4 Molnar, P., Boos, W. R. & Battasti, D. S. Orographic Controls on Climate and Paleoclimate of Asia: Thermal and Mechanical Roles for the Tibetan Plateau. *Annual Review of Earth and Planetary Sciences* **38**, 77–102 (2010).

5 Renner, S. S. Available data point to a 4-km-high Tibetan Plateau by 40Ma, but 100 molecular-clock papers have linked supposed recent uplift to young node ages. *Journal of Biogeography* **43**, 1479–1487 (2016).

6 Schmidt, J., Opgenoorth, L. & Miehe, G. Speciation, uplift, and climate change in *Nepal. An introduction to the natural history, ecology and human environment in the Himalayas. A companion to the Flora of Nepal* (eds G. Miehe & C. Pendry) 168–173 (Royal Botanic Garden Edinburgh, 2016).

7 van Hinsbergen, D. J. *et al.* Greater India Basin hypothesis and a two-stage Cenozoic collision between India and Asia. *Proc Natl Acad Sci U S A* **109**, 7659–7664 (2012).

8 Matthews, K. J., Müller, R. D. & Sandwell, D. T. Oceanic microplate formation records the onset of India–Eurasia collision. *Earth and Planetary Science Letters* **433**, 204–214 (2016).

9 Ma, Y. *et al.* Early Cretaceous paleomagnetic and geochronologic results from the Tethyan Himalaya: Insights into the Neotethyan paleogeography and the India-Asia collision. *Scientific reports* **6**, 21605 (2016).

10 Hetzel, R. *et al.* Peneplain formation in southern Tibet predates the India-Asia collision and plateau uplift. *Geology* **39**, 983–986 (2011).

11 Wang, C. *et al.* Constraints on the early uplift history of the Tibetan Plateau. *Proceedings of the National Academy of Sciences* **105**, 4987–4992 (2008).

12 Rowley, D. B. & Currie, B. S. Palaeo-altimetry of the late Eocene to Miocene Lunpola basin, central Tibet. *Nature* **439**, 677–681 (2006).

13 Ding, L. *et al.* The Andean-type Gangdese Mountains: Paleoelevation record from the Paleocene–Eocene Linzhou Basin. *Earth and Planetary Science Letters* **392**, 250–264 (2014).

14 Tada, R., Zheng, H. & Clift, D. Evolution and variability of the Asian monsoon and its potential linkage with uplift of the Himalaya and Tibetan Plateau. *Progress in Earth and Planetary Science* **3**, 1–26 (2016).

15 Royden, L., Burchfiel, B. C. & van der Hilst, R. D. The Geological Evolution of the Tibetan Plateau. *Science* **321**, 1054–1058 (2008).

16 Rowley, D. B. & Garzione, C. N. Stable Isotope-Based Paleoaltimetry. *Annual Review of Earth and Planetary Sciences* **35**, 463–508 (2007).

17 Tremblay, M. M. *et al.* Erosion in southern Tibet shut down at approximately 10 Ma due to enhanced rock uplift within the Himalaya. *Proceedings of the National Academy of Sciences* **112**, 12030–12035 (2015).

18 Clift, P. D. *et al.* Correlation of Himalayan exhumation rates and Asian monsoon intensity. *Nature Geoscience* **1**, 875–880 (2008).

19 Schleich, H. H. & Kästle, W. *Amphibians and Reptiles of Nepal*. (A.R.G. Gantner Verlag, 2002).

20 Frost, D. R. *Amphibian species of the world: an online reference. Version 5.2 (15 July 2008). Electronic Database*, <http://research.amnh.org/herpetology/amphibia/index.php> (2008).

21 Subba, B., Ravikanth, G. & Aravind, N. A. Scaling new heights: first record of Boulenger's Lazy Toad *Scutiger boulengeri* (Amphibia: Anura: Megophryidae) from high altitude lake in Sikkim Himalaya, India. *Journal of Threatened Taxa* **7**, 7655–7663 (2015).

22 Hjarding, A., Tolley, K. A. & Burgess, N. D. Red List assessments of East African chameleons: a case study of why we need experts. *Oryx*, 1–7 (2014).

23 Newbold, T. Applications and limitations of museum data for conservation and ecology, with particular attention to species distribution models. *Progress in Physical Geography* **34**, 3–22 (2010).

24 Graham, C. H., Ferrier, S., Huettman, F., Moritz, C. & Peterson, A. T. New developments in museumbased informatics and applications in biodiversity analysis. *Trends in Ecology and Evolution* **19**, 497–503 (2004).

25 Chen, W., Bi, K. & Fu, J. Frequent mitochondrial gene introgressionamong high elevation Tibetan megophryid frogs revealed by conflicting genegenealogies. *Mol Ecol* **18**, 2856–2876 (2009).

26 Dubois, A. Miscellanea taxinomica batrachologica (I). *Alytes* **5**, 19 (1987).

27 Sarania, B., Kumar, A., Wang, K. & Rakshit, K. A record of *Scutiger nyingchiensis* Fei, 1977 (Amphibia: Anuran: Megophryidae) in the Eastern Himalaya, North East India. *Current Science India* **109**, 413–414 (2015).

28 Jiang, K. *et al.* A new species of the genus *Scutiger* (Anura: Megophryidae) from southeastern Tibet, China. *Zootaxa* **3388**, 29–40 (2012).

29 Jiang, K. *et al.* A new species of the genus *Scutiger* (Anura: Megophryidae) from Medog of southeastern Tibet, China. *Zoological Research* **37**, 21–30 (2016).

30 Delorme, M. & Dubois, A. Une nouvelle espèce de Scutiger du Bhutan, et quelques remarques sur la classification subgénérique du genre *Scutiger* (Megophryidae, Leptobrachiinae). *Alytes* **19**, 141–153 (2001).

Supplementary Information S3

Scutiger occidentalis Dubois, 1978

Type locality: "un torrent sous le village de Shukdhari, 2920-2940 m, près de Sonamarg, Cachemire" (i.e. "a stream below the village Shukdhari, 2920-2940 m a.s.l., near Sonamarg, Kashmir"), Jammu and Kashmir, India.

Holotype: MNHNP 1977.1069, by original designation (Dubois, 1978).

According to "Lost Species of India" (http://www.lostspeciesindia.org/LAI2/wanted.php) this species has been last seen in India 1977, i.e. from the original description based on 89 specimens. According to Dubois (1978), prior to its description, this taxon has been reported under different scientific names by several authors. Our genetic data from the Deosai Plateau (W-Kashmir, Pakistan) provide evidence that this taxon is a valid species and does not present a junior subjective synonym of *Scutiger nyingchiensis* Fei, 1977, as Dubois (1980, 1987) suggested. Here, we summarize known facts and provide new information on morphological characters and the currently known range of *S. occidentalis*. Figure S3.1 presents defined records for *S. occidentalis* as reported by Dubois (1978), Ficetola et al. (2010), and in the present study. However, note that some of the historic records might be doubtful. As mentioned in the main text, Ficetola in 2006 (Ficetola et al. 2010) and Stöck in 2008 (unpublished, Fig. S3.2) independently found *S. occidentalis* on the Deosai Plateau.

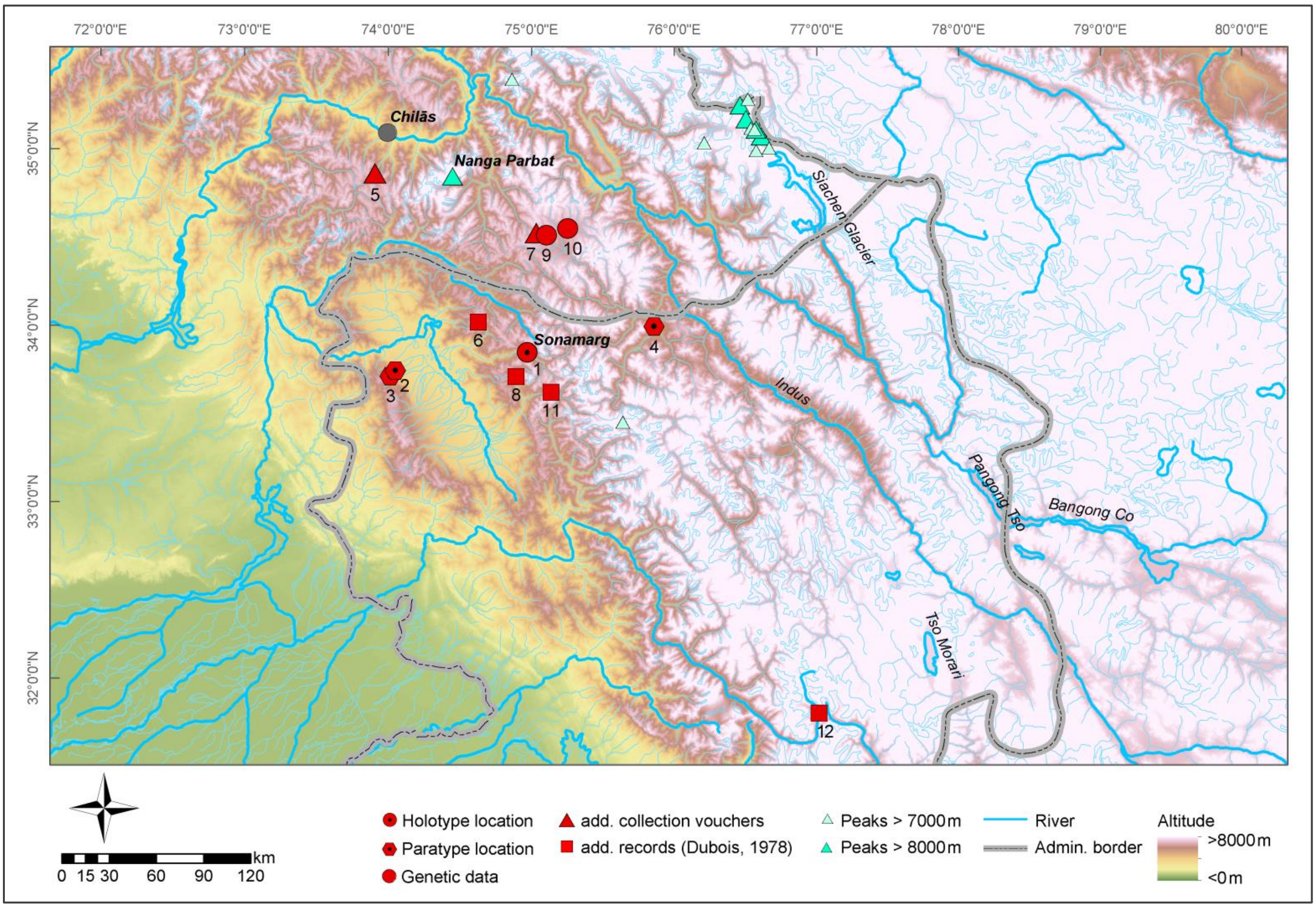

Fig. S3.1. Records of *Scutiger occidentalis*. Apart from the holotype's and paratypes' locations (nos. 1 and 2-4) additional (add.) records, listed in Dubois (1978) or available from collection vouchers, are included. Localities nos. 9 and 10 refer to the records of Ficetola (2010) and Stöck (unpubl.), genetically characterized by the present study; Table S3.1 for details. The map was created using ArcMap 10.3.1 (https://www.esri.com/).

Table S3.1. Records of *Scutiger* (cf.) *occidentalis* as shown in Fig. S3.1. Most coordinates (*) are approximations from verbal descriptions in the original publications. Coordinates are given in decimal degree to match with the format of the original records (9 and 10).

No	long	lat	Altitude	Country	Taxon	Leg. / Reference	Sample-ID / Museum Vouchers	Comment	Description of Location
*1	75.29	34.30	2920-2940	Jammu Kashmir	*S. occidentalis*	Dubois & Vidal (Dubois 1978)	MNHN 1977.1069	holotype	Sonamarg, Cashmer (Jammu & Cashmer, India)
*2	74.37	34.04	2680-2850	Kashmir	*S. occidentalis*	Dubois & Vidal (Dubois 1978)	MNHN 19771129-1144	paratype	between Gulmarg and Khilanmarg, Cashmer
*3	74.38	34.04	2690	Kashmir	*S. occidentalis*	Dubois & Vidal (Dubois 1978)	MNHN 1977.1145	paratype	Gulmarg, Cashmer
*4	76.13	34.55	3660	Ladakh	*S.* cf. *occidentalis*	Boulenger; reference: Duda & Sahi (1977), (Dubois 1978)	BMNH 1919.8.11.44	as *Cophophryne sikkimensis*	Kargil, Ladakh
*5	74.04	35.18	?	Pakistan	*S. occidentalis*	Auffenberg & Rehman	FLMNH 82455, 82456, 82394 to 82407, 82409	museum vouchers	4.2 km N Babusar, 30.7 km S Chilãs
*6	74.92	34.43	3570	Jammu Kashmir	*S.* cf. *occidentalis*	Annandale 1917, (Dubois 1978)		considered *S. occidentalis* by Dubois (1978)	Gangbal Lake
*7	75.22	34.98	?	Pakistan	*S. occidentalis*	Shamin, Fakhri	FLMNH 84663 to 84667	museum vouchers	Deosai Plateau, Sheosar Lake, SW side
*8	75.23	34.15	2740	Kashmir	*S.* cf. *occidentalis*	Annandale 1917 (Dubois 1978)		considered *S. occidentalis* by Dubois (1978)	Lidarwart
9	75.3000	34.9833	4300	Pakistan	*S. occidentalis*	Ficetola in 2006 (Ficetola et al. 2010)	MS_PK1-5	genetic data, photo vouchers	Pakistan, Deosai Plateau
10	75.4400	35.0400	4300	Pakistan	*S. occidentalis*	Stöck in 2008 (this study)	MS_PK6	genetic data, photo vouchers	Pakistan, Deosai Plateau
*11	75.4971	34.0943	3660	India	*S.* cf. *occidentalis*	Ahmad 1946 (Dubois 1978)	-	considered *S. occidentalis* by Dubois (1978)	Shesh Nag lake
*12	77.6171	32.4752	3900	Himachal Pradesh (India)	*S.* cf. *occidentalis*	Ahmad 1946 (Dubois 1978)	-	considered *S. occidentalis* by Dubois (1978)	Chandra Sar Lake
-			3050-3200	no detaiils provided	*S.* cf. *occidentalis*	Annandale 1917 (Dubois 1978)	-	considered *S. occidentalis* by Dubois (1978)	Nagabera
-			3680	no details provided	*S.* cf. *occidentalis*	Annandale 1917 (Dubois 1978)	-	considered *S. occidentalis* by Dubois (1978)	Kreshen Sar Lake

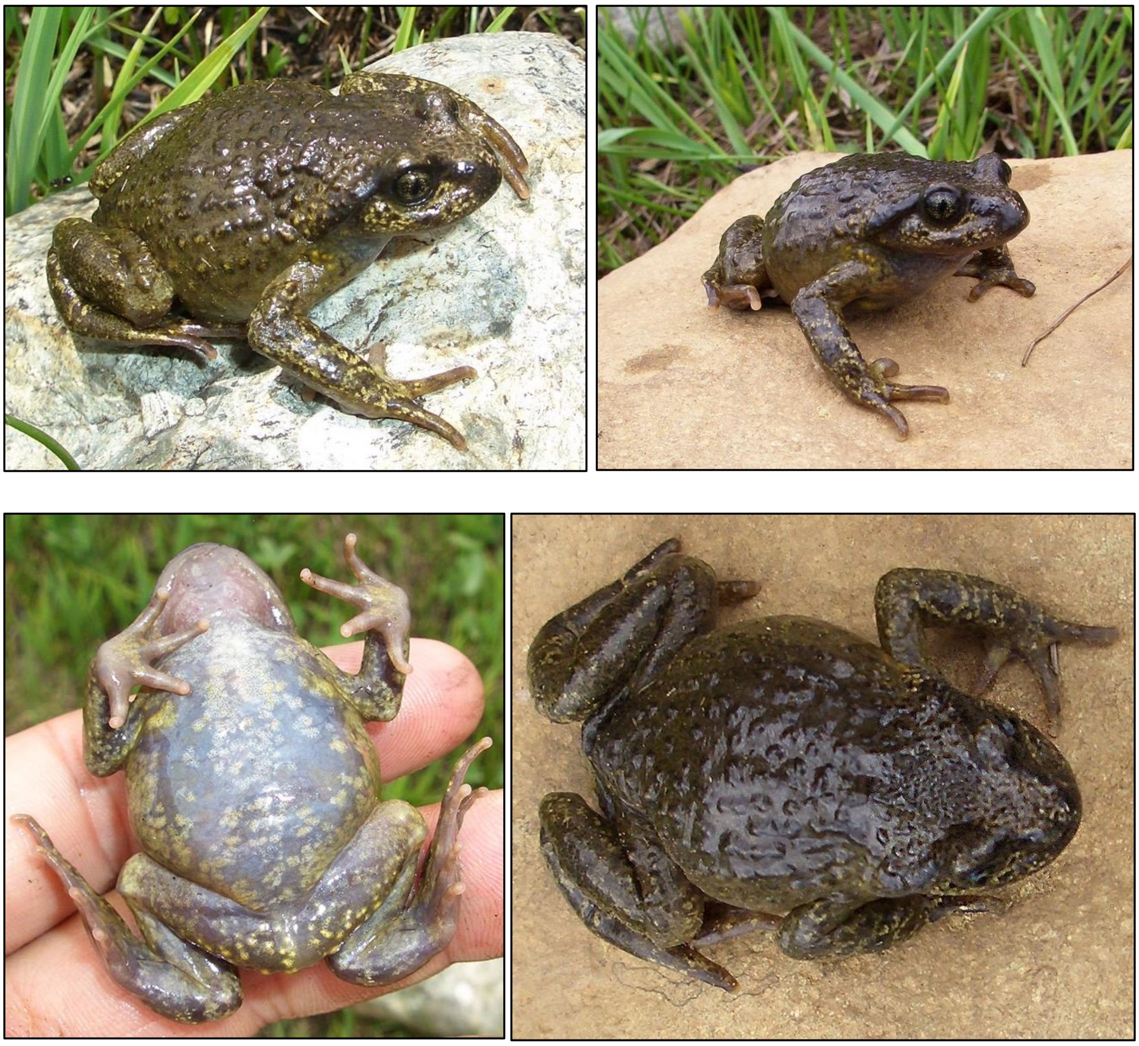

Fig. S3.2. Photo vouchers of *Scutiger occidentalis* (presumably a female, sample ID: MS_PK6) from the present study, sampled for DNA (buccal swabs) on the Deosai Plateau, 4300 m a.s.l., in 2008 (Photographs: M. Stöck).

Here, we further report on two other series of scientific vouchers that we have recently located in the Division of Herpetology of the Florida Museum of Natural History. One large series of specimens (FLMNH 82455, 82456, 82394 to 82407, 82409) represents differently aged specimens, reaching from 15 mm to 65 mm snout-vent-length, and essentially comprises the currently most western known records of *S. occidentalis* from locality no. 5 in Fig. S3.1 (4.2 km N of Babusar, 30.7 km S of Chilas, Pakistan), collected by W. Auffenberg and H. Rehman in 1991. This record exceeds the known range to the SW of the Nanga Parbat Massif. Another series from the FLMNH (84663 to 84667) were collected by F. Shamin from the southwest of the Deosai Plateau (Sheosar Lake), from locality no. 7 in Fig. S3.1.

We depict both series in part in Figs. S3.3 and S3.4. They exemplify that this species belongs to the large *Scutiger* species and gives an impression of the morphological variation of the taxon. Although the nuptial pads on the chests of mature male *Scutiger* have been repeatedly used as species-specific characters (e.g. Dubois 1978: Fig. 3d), their variability may be considerable: as an example, we depict them from two males (FLMNH 82396 and 82402), collected from the same locality.

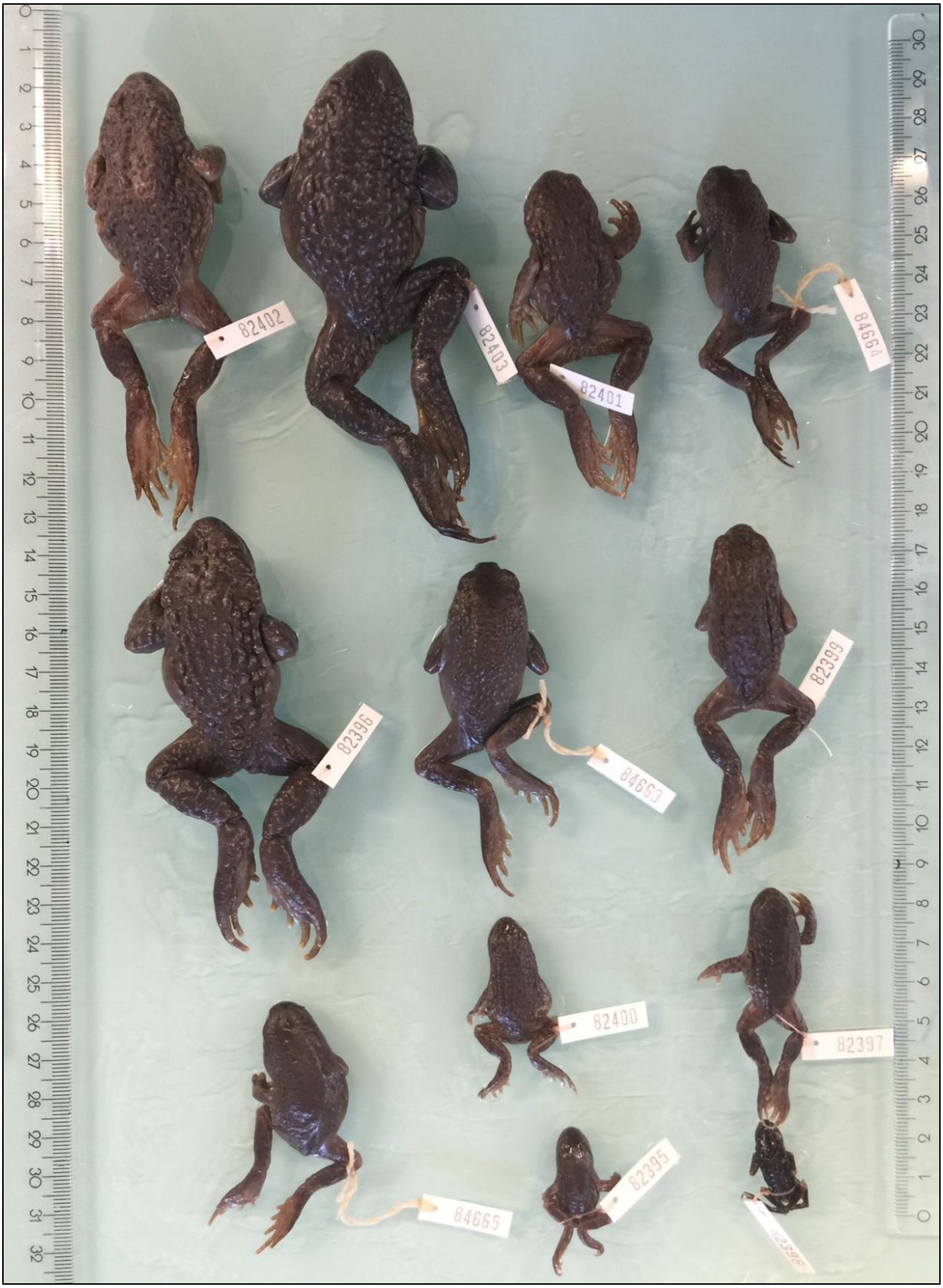

Fig. S3.3. Dorsal view of specimens collected from the FLMNH, see text for details (Photograph: M. Stöck).

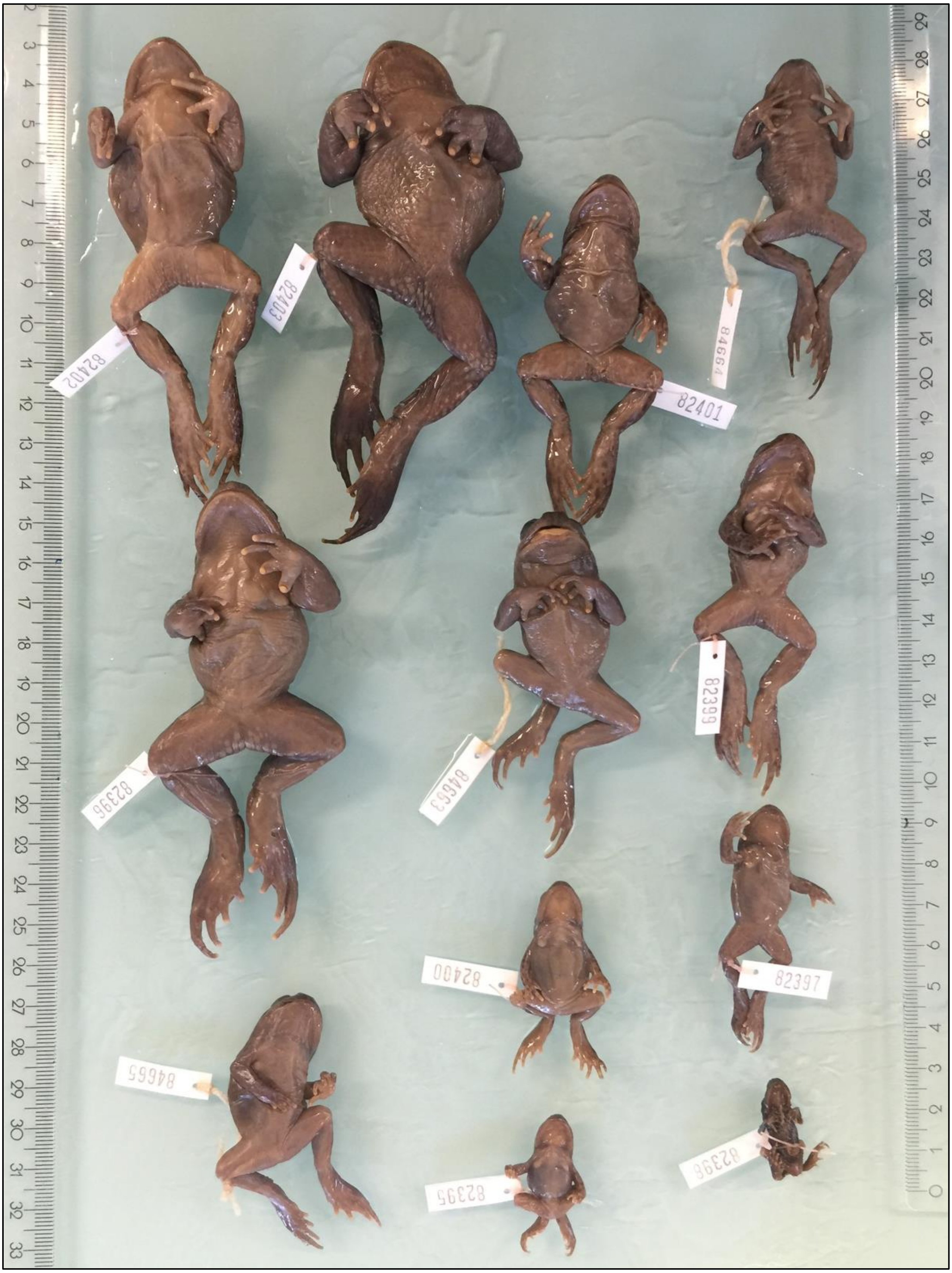

Fig. S3.4. Ventral view of specimens of *Scutiger occidentalis* collected from the FLMNH, see text for details (Photograph: M. Stöck).

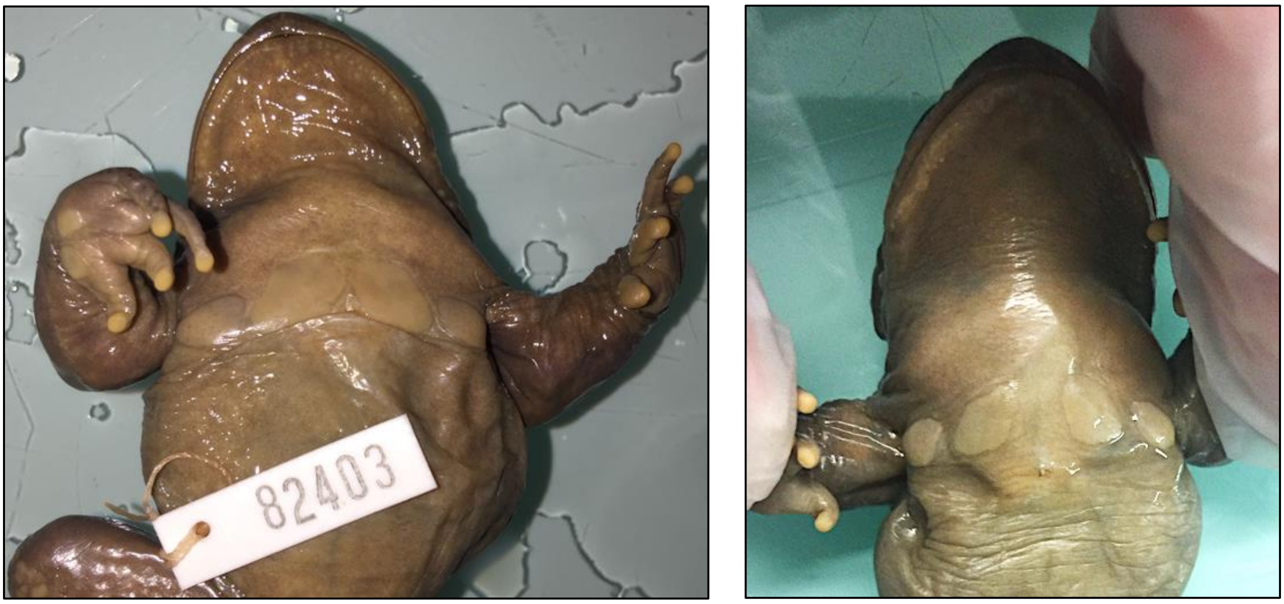

Fig. S3.5. Nuptial pads of two males (left: FLMNH 82403; right: FLMNH 82396) of *S. occidentalis*, out of breeding season. Specimens were collected from the same locality no. 5 in Fig. S3.1 (4.2 km N of Babusar, 30.7 km S of Chilas, Pakistan), by W. Auffenberg and H. Rehman in 1991.

The possibly only known-to-science tadpole

To our knowledge, the tadpoles of *S. occidentalis* have not been known to science through any previous publication. Among the series of specimens, Auffenberg & Rahman collected on 9/9/1991 “under stones in a weed-choked small stream in a wet meadow” at locality no. 5 in Fig. S3.1 (4.2 km N of Babusar, 30.7 km S of Chilas, Pakistan), is an about 70 mm long tadpole (FLMNH 82394). Its oral disc is depicted in Fig. S3.6. Its species identification appears correct since the only other anuran species in this region and elevation belong to members of the *Bufo viridis* subgroup (Stöck et al. 2006) are distinguished by a different oral disc morphology.

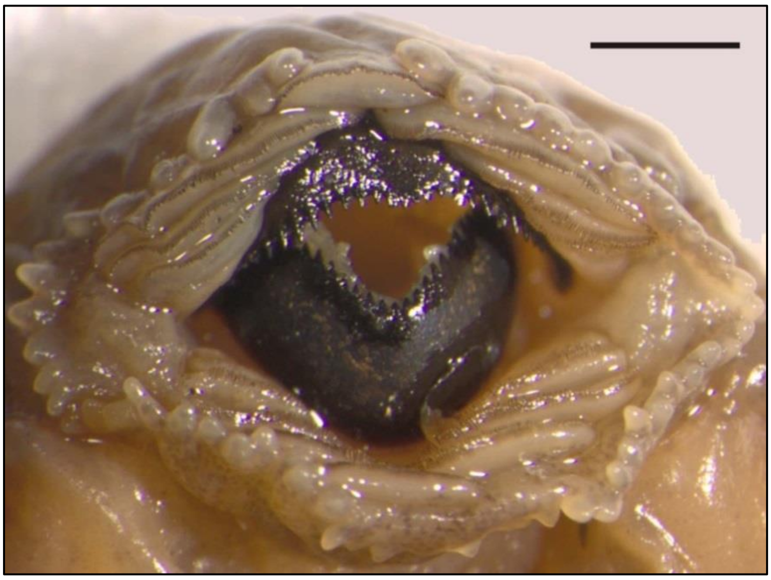

Fig. S3.6. The oral disc and mouth of a fixed tadpole of *Scutiger occidentalis*. Shown is specimen FLMNH 82394, collected by Auffenberg & Rahman, 9/9/1991), total length about 70 mm. The bar represents 1 mm. Photograph: M. Stöck.

References

Dubois, A. Une espèce des *Scutiger* Theobald, 1868 de l'Himalaya occidental (Anura: Pelobatidae). Senckenbergiana biologica **59**, 163–171 (1978).

Dubois, A. Notes sur la systematique et la repartition des amphibiens anoures de Chine et des regions avoisinantes IV. Classification generique et subgenerique des Pelobatidae, Megophryinae. Bulletin Mensuel de la Société Linnéenne de Lyon **49**, 469–482 (1980).

Dubois, A. Miscellanea taxinomica batrachologica (I). Alytes. Paris 5: 7–95 (1987; "1986").

Fei, L. A survey of amphibians in Xizang (Tibet). Acta Zoologica Sinica/ Dong wu xue bao, Beijing **23**, 54–63 (1977).

Ficetola, G. F., A. Crottini, M. Casiraghi, and E. Padoa-Schioppa. New data on amphibians and reptiles of the Northern Areas of Pakistan: distribution, genetic variability and conservation issues. North-Western Journal of Zoology, Oradea, Romania **6**, 1–12 (2010).

Stöck, M., Moritz, C., Hickerson, M., Frynt,,a D., Dujsebayeva T., Eremchenko, V., Macey, J.R., Papenfuss, T. J. & Wake, D. B. (2006): Evolution of mitochondrial relationships and biogeography of Palearctic green toads (*Bufo viridis* subgroup) with insights in their genomic plasticity. Molecular Phylogenetics and Evolution **41**, 663-689.

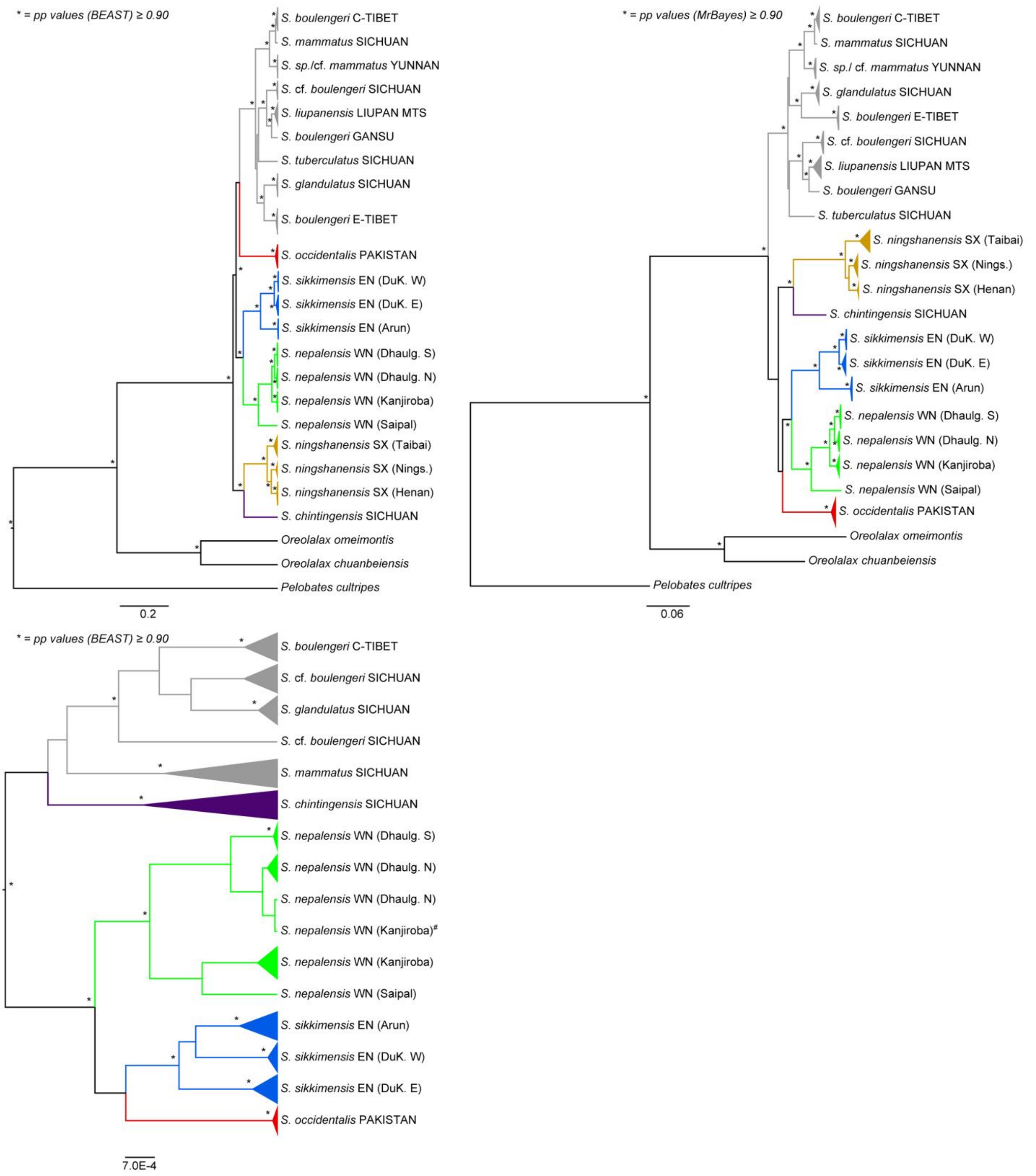

Figure S1. Bayesian trees based on the combined mtDNA (upper panels) and nuDNA sequence data (lower panel) inferred with BEAST and MrBayes. *Scutiger* (*S.*) is followed by the species name and by locality information (Table S5 for sample IDs). Nodes with multiple nodes from the same taxonomic unit and locality, and a posterior probability (pp) value ≥ 0.90 were collapsed. #voucher A2014-13 from Kanjiroba Himal was placed differently in the mtDNA and nDNA tree (for details see main text); C-Tibet=Central Tibet; Dhaulag. N/S=Dhaulagiri Himal North/South; DuK. E/W=Dudh Koshi River East/West; E-Tibet=East Tibet; EN=East Nepal; MTS=Mountains; Nings.=Ningshan; SX=Shaanxi; WN=West Nepal.

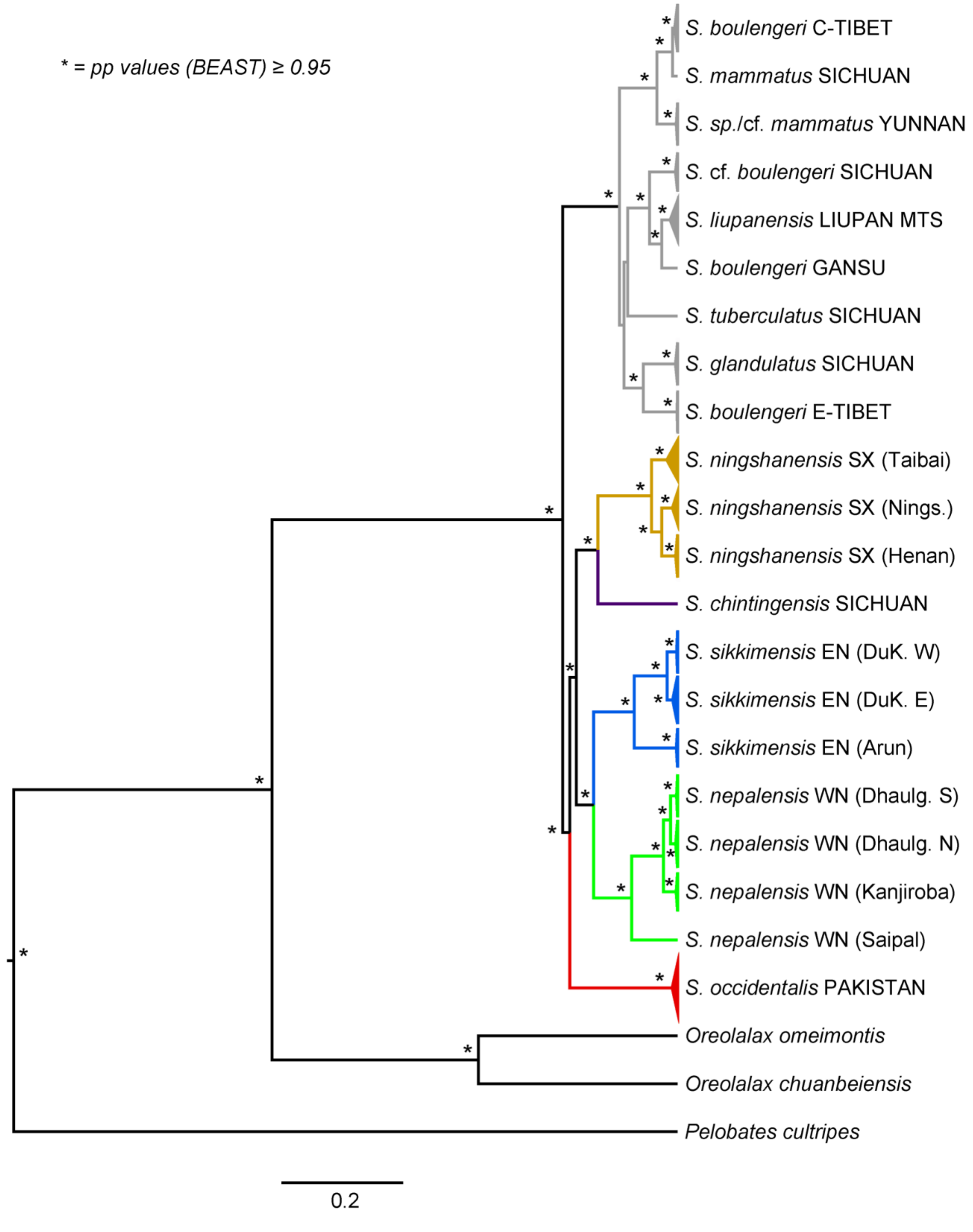

Figure S2. Backbone constraint tree of the preferred model m3.3 inferred with BEAST. *Scutiger* (*S.*) is followed by the species name and by locality information (Table S5 for sample IDs). Nodes with multiple nodes from the same taxonomic unit and locality, and a posterior probability (pp) value ≥ 0.95 were collapsed. C-Tibet=Central Tibet; Dhaulag. N/S=Dhaulagiri Himal North/South; DuK. E/W=Dudh Koshi River East/West; E-Tibet=East Tibet; EN=East Nepal; MTS=Mountains; Nings.=Ningshan; SX=Shaanxi; WN=West Nepal.

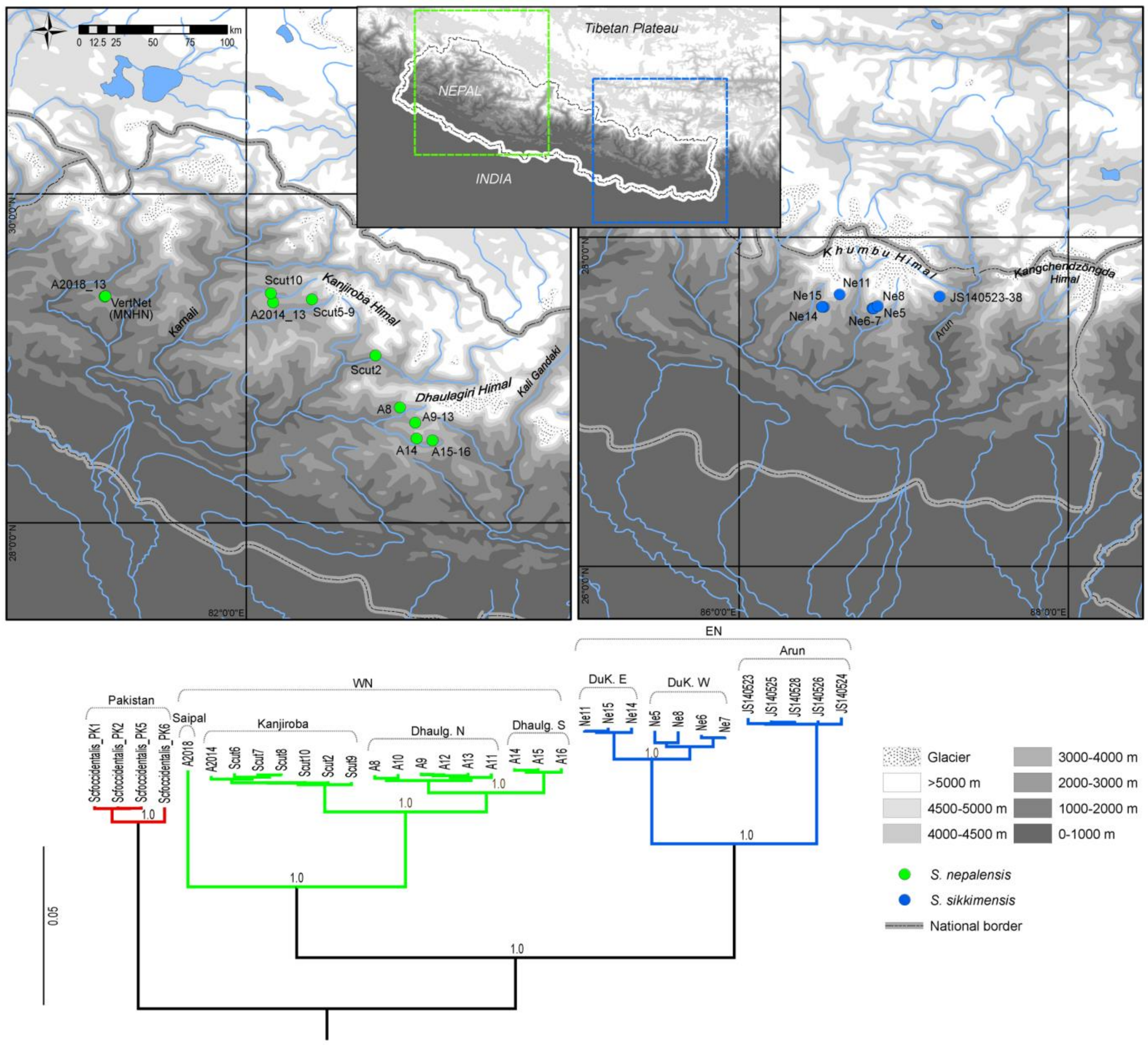

Figure S3. Comparison of mtDNA phylogeny and sampling sites for *Scutiger nepalensis* (green; left panel) and *S. sikkimensis* (blue; right panel). The tree was inferred by Bayesian Inference analysis using MrBayes v3.2.6 [1] based on concatenated mtDNA sequences; we run 10 million generations with four chains, starting with a random tree, sampling trees every 1000th generation until reaching an average standard deviation of split frequencies < 0.01. Partition scheme and substitution models were selected with PartitionFinder 1.1.1 [2]. Colour codes match those in Figs. 1-3. The record “VertNet (MNHN)” refers to vouchers of *S. nepalensis* from “Khaptar”, available via VertNet (http://vertnet.org/) and deposited at Museum National d'Histoire Naturelle Paris (MNHN), France. Dhaulg. N/S=Dhaulagiri Himal North/South; DuK. E/W=Dudh Koshi River East/West; EN=East Nepal; WN=West Nepal. Map created in ArcMap 10.3.1 (https://www.esri.com/).

References

1. Ronquist, F. et al. MrBayes 3.2: efficient Bayesian phylogenetic inference and model choice across a large model space. Systematic Biology **61**, 539–542 (2012).

2. Lanfear, R., Calcott, B., Ho, S. Y. & Guindon, S. PartitionFinder: combined selection of partitioning schemes and substitution models for phylogenetic analyses. Molecula Biology and Evolution **29**, 1695–1701 (2012).

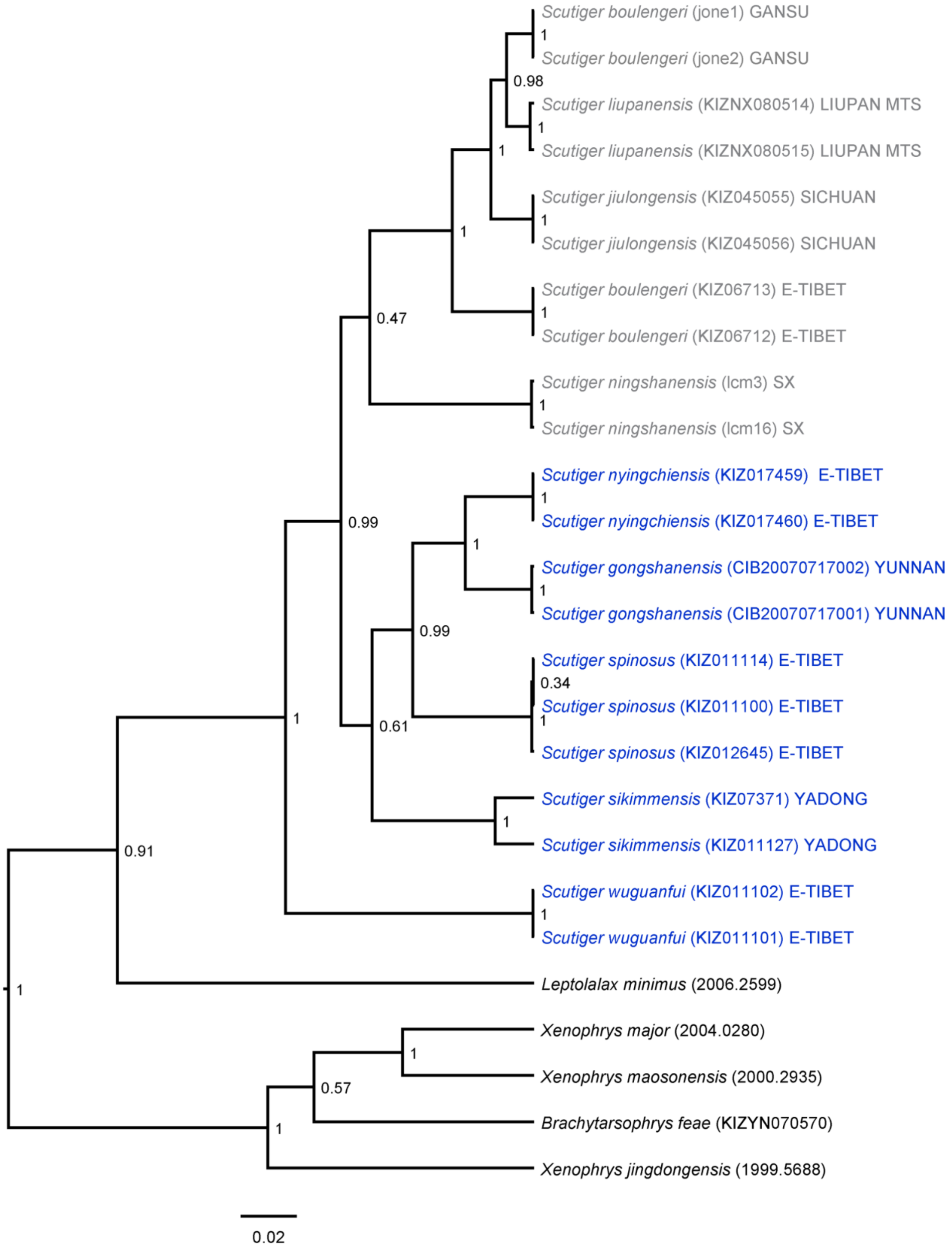

Figure S4. Bayesian inference tree based on *co1* sequences of the dataset as used in Jiang et al. 2016 [1]. Species name is followed by accession or sample number and by locality information. Species from the southern slope of the Himalaya are in blue, while those from the Tibet & Hengduan Shan area are in grey letters. E-Tibet=East Tibet; Liupan Mts=Liupan Mountains; SX=Shaanxi.

Reference

Jiang, K. et al. A new species of the genus *Scutiger* (Anura: Megophryidae) from Medog of southeastern Tibet, China. Zoological Research **37**, 21–30 (2016).

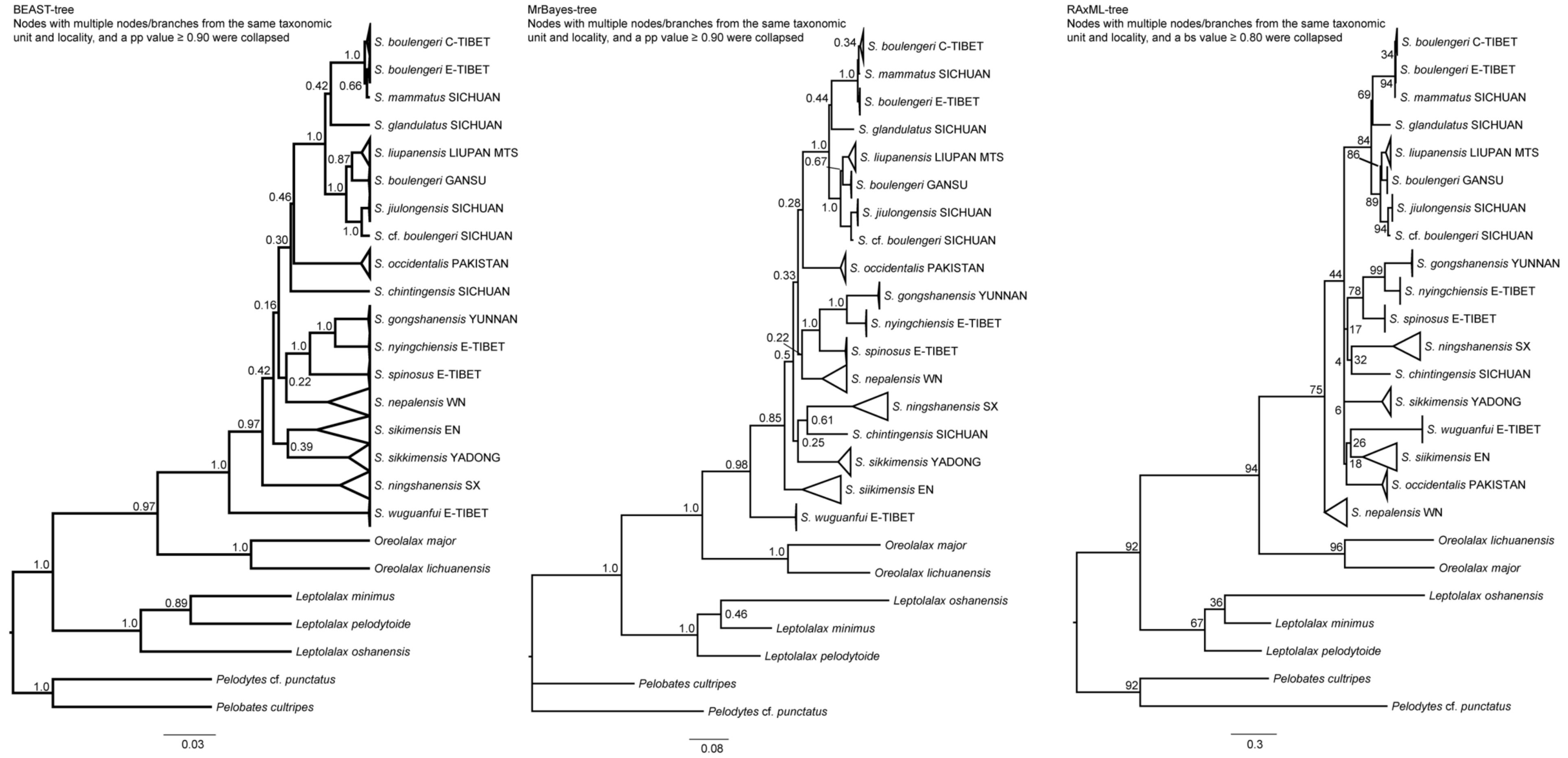

Figure S5. Bayesian inference trees (left, middle) and Maximum likelihood tree (right) based on *co1* sequences of the dataset as used in Jiang et al. 2016 extended by sequences of this study and from NCBI. *Scutiger* species name is followed by locality information (Table S5 for sample IDs). C-Tibet=Central Tibet; EN=East Nepal; E-Tibet=East Tibet; Liupan Mts=Liupan Mountains; SX=Shaanxi; WN=West Nepal.

Table S1. Summarized results of the topological tests of different phylogenetic hypothesis (PH) for the mitochondrial data set. Approximately unbiased (AU), Shimodaira–Hasegawa (SH) and weighted SH (WSH) test (p-values) were performed using CONSEL[1], while marginal likelihoods for the Bayes Factor (BF) model selection approach were estimated based on the stepping stone (ss) and path sampling (ps) method in BEAST v.1.8.4[2,3]. A=Tibet-Hengduan Shan clade; B=*S. chintingensis*/ *S. ningshanensis* (Tsinling Mountains & Sichuan Basin clade); C=*S. nepalensis*/*S. sikkimensis* (Himalayan clade); D=*S. occidentalis*. Options that resampled topologies of other models were not tested (marked in italics). Best-supported model is in bold. A 2lnBF = 0–2 means "not worth more than a bare mention", 2lnBf = 2–6 means "positive" support, 2lnBf = 6–10 provides "strong" support, and 2lnBf >10 means "decisive" support.

Model	*PH*	*Remark*	*AU*	*SH*	*WSH*	*ss*	*ps*	*2lnBF*	
								ss	*ps*
m1	(B(C(AD)))	mt-BEAST tree	0.501	0.810	0.830	-18901.4	-18902.9	m1:m2=3.4	m1:m2=4.4
m2	(A(B(CD)))	mt-MrBayes tree	0.212	0.507	0.651	-18903.1	-18900.7	m1:m3.1=150.4	m1:m3.1=152.4
m3.1	(D(ABC))	mt-ML tree (v1)	0.635	0.869	0.868	-18976.6	-18979.1	m1:m3.3=29.4	m1:m3.3=34.6
m3.2	*(B(ACD))*	*mt-ML tree (v2) ≙m1*						m1:m3.5=25.0	m1:m3.5=26.0
**m3.3**	(A(D(BC)))	mt-ML tree (v3)	0.392	0.813	0.795	**-18886.7**	**-18885.5**	m1:m3.7=4.6	m1:m3.7=5.2
m3.4	*(A(B(CD)))*	*mt-ML tree (v4) ≙m2*						m1:m3.8=5.2	m1:m3.8=3.4
m3.5	((AD)(BC))	mt-ML tree (v5)	0.378	0.822	0.856	-18890.6	-18887.7	m1:m4=74.4	m1:m4=79.8
m3.6	*((BA)(CD))*	*mt-ML tree (v6) ≙m4*						m2:m3.1=147.0	m2:m3.1=156.8
m3.7	(A((C(BD))	mt-ML tree (v7)	0.293	0.633	0.711	-18903.7	-18905.5	m2:m3.3=32.8	m2:m3.3=30.2
m3.8	(C(A(BD)))	mt-ML tree (v8)	0.553	0.696	0.721			m2:m3.5=172.0	m2:m3.5=182.8
m4	((BA)(CD))	nu-trees	0.172	0.470	0.576	-18938.6	-18942.8	m2:m3.7=1.2	m2:m3.7=9.6
								m2:m3.8=8.6	m2:m3.8=1.0
								m2:m4=71.0	m2:m4=84.2
								m3.1:m3.3=179.8	m3.1:m3.3=187.0
								m3.1:m3.5=172.0	m3.1:m3.5=182.8
								m3.1:m3.7=145.8	m3.1:m3.7=147.2
								m3.1:m3.8=155.6	m3.1:m3.8=155.8
								m3.1:m4=76.0	m3.1:m4=72.6
								**m3.3:m3.5=7.8**	**m3.3:m3.5=4.2**
								m3.3:m3.7=34.0	m3.3:m3.7=39.8
								m3.3:m3.8=24.2	m3.3:m3.8=31.2
								m3.3:m4=103.8	m3.3:m4=114.4
								m3.5:m3.7=26.2	m3.5:m3.7=35.6
								m3.5:m3.8=16.4	m3.5:m3.8=27.0
								m3.5:m4=96.0	m3.5:m4=110.2
								m3.7:m3.8=9.8	m3.7:m3.8=8.6
								m3.7:m4=69.8	m3.7:m4=74.6
								m3.8:m4=79.6	m3.8:m4=83.2

1. Shimodaira, H. & Hasegawa, M. CONSEL: for assessing the confidence of phylogenetic tree selection. Bioinformatics 17, 1246–1247, (2001).
2. Drummond, A. J. & Rambaut, A. BEAST: Bayesian evolutionary analysis by sampling trees. BMC Evolutionary Biology 7, 214, (2007).
3. Drummond, A. J. et al. Bayesian phylogenetics with BEAUti and the BEAST 1.7. Molecular Biology and Evolution 29, 1969–1973, (2012).

Table S2. Genetic between-group mean distances among *Scutiger* species based on concatenated four mitchondrial (a) and three nuclear (b) sequence data. Lower matrix: p-distances, upper matrix: SD. *Scutiger sp*. and *S*. cf. *mammatus* refer to specimens from Yunnan [1].

(a) Scutiger	*boulengeri*	*glandulatus*	*nepalensis*	*occidentalis*	*sikkimensis*	*liupanensis*	*ningshanensis*	*sp.*	*cf. mammatus*	*mammatus*	*chintingensis*	*tuberculatus*
boulengeri (n=20)		0.37	0.46	0.46	0.51	0.47	0.76	0.36	0.35	0.28	0.46	0.45
glandulatus (n=6)	6.60		0.53	0.52	0.51	0.63	0.87	0.46	0.46	0.42	0.49	0.57
nepalensis (n=17)	9.72	9.72		0.50	0.46	0.75	0.78	0.53	0.53	0.48	0.53	0.66
occidentalis (n=4)	10.10	9.84	9.91		0.50	0.77	0.86	0.58	0.58	0.47	0.53	0.74
sikkimensis (n=12)	11.17	11.01	9.19	10.07		0.83	0.81	0.59	0.59	0.52	0.53	0.74
liupanensis (n=8)	6.15	6.66	11.35	11.00	12.86		0.90	0.87	0.82	0.68	0.71	0.81
ningshanensis (n=15)	13.38	13.57	13.59	13.16	13.78	13.44		1.06	1.06	0.78	0.67	0.99
sp. (n=1)	5.40	6.15	8.96	9.74	10.52	8.06	13.01		0.10	0.34	0.62	0.56
cf. *mammatus* (n=1)	5.26	6.00	8.85	9.60	10.42	7.76	12.77	0.23		0.33	0.61	0.55
mammatus (n=1)	4.71	6.32	9.67	9.74	10.92	7.77	12.85	3.04	2.90		0.52	0.56
chintingensis (n=1)	9.75	9.60	9.39	9.42	10.14	11.05	10.93	9.01	8.92	9.31		0.67
tuberculatus (n=1)	6.03	5.37	8.92	9.37	10.31	7.50	13.03	5.34	5.14	5.61	8.71	

(b) Scutiger	*nepalensis*	*sikkimensis*	*boulengeri*	*glandulatus*	*occidentalis*	*mammatus*	*chintingensis*
nepalensis (n=14)		0.11	0.22	0.25	0.13	0.17	0.17
sikkimensis (n=12)	0.7		0.22	0.24	0.12	0.18	0.16
boulengeri (n=16)	1.2	1.3		0.16	0.20	0.17	0.22
glandulatus (n=6)	1.5	1.5	0.7		0.24	0.22	0.25
occidentalis (n=2)	0.7	0.6	1.0	1.3		0.16	0.15
mammatus (n=2)	1.1	1.1	0.9	1.3	0.8		0.15
chintingensis (n=3)	1.0	1.0	1.2	1.4	0.7	0.9	

Reference

Rao, D.-Q. & Wilkinson, J. A. Phylogenetic relationships of the mustache toads inferred from mtDNA sequences. Molecular Phylogenetics and Evolution **46**, 61–73 (2008).

Table S3. Age estimates (in million years) for each node referenced in Figures 3. Estimates (div. time) are given in millions of years, with the 95% highest posterior density interval (HDPI). This study=divergence time estimation based on node constraints obtained from fossil records and estimated ages in previous studies [1-4].

	**This study**		**Pyron 2014**	**Zhang et al. 2013**				**Roelants et al. 2007**		**San Mauro et al. 2005**	
Node no	***div. time***	***95% HPDI***	***div. time (r8s)***	***div. time (MDT)***	***95% CI***	***div. time (BEAST)***	***95% CI***	***div. time***	***95% CI***	***div. time***	***95% CI***
1	209.5	193.6-227.9	209.5	209.0	186-238	236.8	199-278	233.9	210.2-258.2	na	na
2	193.3	171.3-215.0	204.8	204.6	182-223	229.8	192-269	228.8	204-253	na	na
3	180.1	154.5-209.3	160.1	191.0	168-219	207.6	154-266	202.9	179.6-228.3	198.6	155.3-243.5
4	171.7	158.0-191.9	190.4	180.4	159-207	178.3	151-219	193.3	169.6-217.5	na	na
5	157.6	135.7-181.3	155.7	158.1	137-183	145.7	105-194	167.3	145.5-190.9	163.6	121.4-208.5
6	152.1	na#	110.1	168.1	146-194	185.1	117-247	151.2	128.8-179.2	151.5	104.8-199.4
7	135.5	115.2-157.2	142.7	146.6	126-171	127.1	89-171	143.1	120.8-166.1	142.5	100.5-186.6
8	109.3	83.9-132.3	125.5	124.1	104-148	102	61-136	114.6	93.2-135.9	117.9	77.2-161.8
9*	89.4	66.7-110.1	96.8	102.6	84-125	79.3	47-110	63.8	49.4-80.3	na	na
10	64.2	47.5-81.4	57.5	na	na	na	na	na	na	na	na
11	53.4	39.4-70.5	48.7	na	na	na	na	na	na	na	na
12	29.2	20.4-38.6	na	na	na	na	na	na	na	na	na
13	28.8	14.9-44.0	na	na	na	na	na	na	na	na	na
14	25.5	18.2-34.6	na	na	na	na	na	na	na	na	na
15	22.8	15.4-31.1	na	na	na	na	na	na	na	na	na
16	20.4	13.6-28.6	na	na	na	na	na	na	na	na	na
17	18.0	11.2-26.9	na	na	na	na	na	na	na	na	na
18	12.1	8.7-16.1	6.3	na	na	na	na	na	na	na	na
19	11.7	6.3-18.3	na	na	na	na	na	na	na	na	na
20	10.9	5.6-17.5	na	na	na	na	na	na	na	na	na
21	10.1	6.7-13.7	na	na	na	na	na	na	na	na	na
22	7.0	3.9-11.0	na	na	na	na	na	na	na	na	na
23	6.0	3.1-9.5	na	na	na	na	na	na	na	na	na
24	5.5	2.8-9.0	na	na	na	na	na	na	na	na	na
25	4.1	1.5-7.4	na	na	na	na	na	na	na	na	na
26	4.0	1.8-6.9	na	na	na	na	na	na	na	na	na
…	<4		na	na	na	na	na	na	na	na	na

*Megophryidae [5]; #no HPD intervals are presented, because the node occur in less than 50% of the trees.

1. Pyron, R. A. Biogeographic analysis reveals ancient continental vicariance and recent oceanic dispersal in amphibians. Systematic Biology **63**, 779–797 (2014).
2. Zhang, P. et al. Efficient sequencing of Anuran mtDNAs and a mitogenomic exploration of the phylogeny and evolution of frogs. Molecular Biology and Evolution **30**, 1899–18915 (2013).
3. Roelants, K. et al. Global patterns of diversification in the history of modern amphibians. Proceedings of the National Academy of Sciences **104**, 887–92 (2007).
4. San Mauro, D. et al. Initial diversification of living amphibians predated the breakup of Pangaea. American Naturalist. **165**, 590–9 (2005).
5. Frost, D. R. et al. The amphibian tree of life. Bulletin of the American Museum of Natural History **297**, 1–370 (2006).

Table S4. Estimates of evolutionary divergence (uncorrected p-distances) based on partial *co1* (556 bp) between haplotypes of major lineages from *Scutiger* species ([1-4], our samples). Species name is followed by sample name (own data) or GenBank accession number and location. Conspecific distances (≤ 2.5 %) are light grey-shaded, while intraspecific distances >2.5 % are dark grey-shaded. Divergences between *S. occidentalis* and *S. nyingchiensis* are in bold. C = Central; E = East; W = West, NH = Ningxia Hui.

		[1]	[2]	[3]	[4]	[5]	[6]	[7]	[8]	[9]	[10]	[11]	[12]	[13]	[14]	[15]	[16]	[17]	[18]	[19]	[20]	[21]	[22]	[23]	[24]	[25]	[26]	[27]	[28]	[29]	[30]
[1]	*S. boulengeri* (A1AL_2010) C-TIBET		0.3	0.0	0.3	1.1	1.1	1.5	1.1	1.4	1.4	1.1	1.1	1.1	1.1	0.3	1.5	1.5	1.3	1.3	1.3	1.3	1.4	1.5	1.4	1.4	1.5	1.5	1.5	1.5	1.5
[2]	*S. boulengeri* (A5AL_2010) C-TIBET	0.5		0.3	0.3	1.0	1.0	1.4	1.1	1.4	1.4	1.1	1.0	1.0	1.1	0.3	1.5	1.4	1.3	1.3	1.3	1.3	1.4	1.4	1.4	1.4	1.5	1.4	1.5	1.5	1.5
[3]	*S. boulengeri* (JN700837) TIBET	0.0	0.5		0.3	1.1	1.1	1.5	1.1	1.4	1.4	1.1	1.1	1.1	1.1	0.3	1.5	1.5	1.3	1.3	1.3	1.3	1.4	1.5	1.4	1.4	1.5	1.5	1.5	1.5	1.5
[4]	*S. boulengeri* (KU243064) E-TIBET	0.7	0.5	0.7		1.1	1.1	1.4	1.1	1.4	1.4	1.1	1.0	1.1	1.1	0.2	1.5	1.4	1.3	1.3	1.3	1.3	1.4	1.5	1.4	1.4	1.5	1.4	1.5	1.5	1.4
[5]	*S. cf. boulengeri* (KQ8-2014) SICHUAN	7.4	7.2	7.4	7.2		0.8	1.4	1.0	1.4	1.4	0.5	0.7	0.8	0.8	1.0	1.4	1.4	1.3	1.3	1.3	1.3	1.4	1.5	1.4	1.4	1.5	1.4	1.5	1.5	1.4
[6]	*S. boulengeri* (KJ082073) GANSU	7.7	7.6	7.7	7.6	3.8		1.4	1.1	1.5	1.5	0.8	0.7	0.7	0.7	1.1	1.3	1.3	1.3	1.3	1.3	1.4	1.4	1.4	1.4	1.4	1.4	1.5	1.4	1.4	1.3
[7]	*S. chintingensis (LC141)* SICHUAN	13.1	12.6	13.1	12.9	11.9	12.2		1.4	1.5	1.5	1.4	1.4	1.4	1.4	1.4	1.3	1.3	1.4	1.3	1.3	1.4	1.4	1.4	1.3	1.4	1.4	1.4	1.4	1.4	1.4
[8]	*S. glandulatus* (Sc1-2014) SICHUAN	7.6	7.6	7.6	7.6	7.2	7.0	12.1		1.5	1.5	1.0	1.0	1.0	1.1	1.0	1.4	1.4	1.3	1.3	1.3	1.4	1.5	1.5	1.5	1.5	1.4	1.4	1.5	1.4	1.4
[9]	*S. gongshanensis* (KU243062) YUNNAN	14.2	13.7	14.2	14.0	13.3	13.7	14.0	14.4		0.2	1.4	1.4	1.4	1.4	1.4	1.4	1.4	1.4	1.4	1.4	1.1	1.5	1.5	1.4	1.4	1.4	1.5	1.3	1.4	1.3
[10]	*S. gongshanensis* (KU243063) YUNNAN	14.4	13.8	14.4	14.2	13.5	13.8	14.2	14.6	0.2		1.4	1.4	1.4	1.4	1.4	1.4	1.4	1.4	1.4	1.4	1.1	1.5	1.5	1.4	1.4	1.4	1.5	1.3	1.4	1.3
[11]	*S. jiulongensis* (KU243066) SICHUAN	7.6	7.6	7.6	7.6	1.6	4.1	11.9	6.8	14.0	14.2		0.8	0.8	0.9	1.1	1.4	1.4	1.3	1.3	1.3	1.4	1.4	1.4	1.4	1.4	1.4	1.4	1.4	1.4	1.4
[12]	*S. liupanensis* (KC140483) NH	7.9	7.7	7.9	7.7	3.4	2.5	11.5	6.3	13.5	13.7	4.1		0.2	0.5	1.0	1.3	1.3	1.2	1.4	1.3	1.3	1.4	1.4	1.4	1.4	1.4	1.4	1.4	1.4	1.4
[13]	*S. liupanensis* (KC140479) NH	8.1	7.9	8.1	7.9	3.6	2.7	11.3	6.5	13.3	13.5	4.0	0.2		0.5	1.0	1.3	1.3	1.2	1.4	1.3	1.3	1.4	1.4	1.4	1.4	1.4	1.4	1.4	1.4	1.4
[14]	*S. liupanensis* (KC140474) NH	8.3	8.1	8.3	8.1	4.5	3.2	11.3	7.2	13.1	13.3	5.2	1.4	1.6		1.1	1.4	1.3	1.3	1.3	1.3	1.4	1.4	1.4	1.4	1.4	1.5	1.4	1.4	1.4	1.3
[15]	*S. mammatus (LC045)* SICHUAN	0.7	0.5	0.7	0.4	7.2	7.6	12.8	7.6	13.8	14.0	7.6	7.7	7.9	8.1		1.5	1.4	1.3	1.3	1.3	1.3	1.4	1.4	1.4	1.4	1.5	1.4	1.5	1.5	1.5
[16]	*S. nepalensis* (A8-2012) W-NEPAL	13.5	13.3	13.5	13.1	12.9	12.1	11.9	12.4	13.3	13.5	12.6	11.9	12.1	12.2	12.9		0.6	1.1	1.3	1.3	1.3	1.5	1.5	1.4	1.3	1.3	1.3	1.4	1.4	1.3
[17]	*S. nepalensis* (MS_Scut2) W-NEPAL	13.3	13.1	13.3	12.9	12.2	11.3	10.6	12.2	12.8	12.9	12.6	11.0	11.2	11.2	12.8	2.0		1.1	1.3	1.3	1.3	1.4	1.4	1.3	1.3	1.3	1.4	1.4	1.4	1.2
[18]	*S. nepalensis* (A2018/13) W-NEPAL	11.9	11.7	11.9	11.5	11.5	11.0	11.5	12.1	12.9	13.1	11.2	10.8	11.0	11.2	11.3	7.6	7.2		1.3	1.3	1.4	1.5	1.5	1.5	1.4	1.4	1.4	1.5	1.5	1.2
[19]	*S. occidentalis* (MS_Pk2) PAKISTAN	11.0	10.6	11.0	10.8	10.3	10.4	11.2	10.8	13.7	13.8	9.9	10.8	11.0	11.0	10.6	12.2	11.7	11.3		0.5	1.4	1.4	1.4	1.4	1.3	1.3	1.4	1.4	1.4	1.4
[20]	*S. occidentalis* (MS_Pk6) PAKISTAN	11.2	11.2	11.2	11.0	10.8	11.0	11.0	11.0	12.8	12.9	10.4	11.3	11.5	11.5	10.8	11.3	10.8	11.2	1.6		1.3	1.4	1.4	1.3	1.3	1.3	1.4	1.4	1.4	1.3
[21]	*S. nyingchiensis* (KU243056) TIBET	12.6	12.4	12.6	12.6	12.2	12.8	12.8	13.1	7.9	8.1	12.6	12.1	11.9	12.1	12.4	12.2	11.7	12.2	**12.6**	**12.1**		1.3	1.4	1.3	1.3	1.3	1.5	1.4	1.4	1.3
[22]	*S. ningshanensis* (KF757408) SHAANXI	14.9	14.4	14.9	14.6	13.3	13.8	13.3	15.3	14.9	15.1	13.1	13.7	13.5	13.7	14.4	14.9	14.4	15.5	13.3	13.3	13.5		0.7	0.9	0.8	1.5	1.5	1.4	1.5	1.4
[23]	*S. ningshanensis* (KF757419) SHAANXI	15.6	15.1	15.6	15.3	14.0	13.7	13.3	15.6	14.7	14.9	13.8	13.3	13.1	12.9	15.1	15.1	14.7	16.2	14.0	14.0	14.0	3.1		1.0	1.0	1.4	1.5	1.4	1.5	1.4
[24]	*S. ningshanensis* (KF757427) SHAANXI	13.1	12.6	13.1	12.9	12.8	12.9	11.5	13.5	13.3	13.5	12.8	13.3	13.5	13.1	12.8	13.1	12.9	14.4	12.4	11.9	12.8	4.9	6.3		0.6	1.4	1.4	1.4	1.4	1.3
[25]	*S. ningshanensis* (KF757437) SHAANXI	12.6	12.1	12.6	12.4	12.4	12.4	11.7	12.9	13.7	13.8	12.4	12.4	12.6	11.9	12.2	12.1	11.9	13.8	12.2	12.1	12.8	4.5	5.9	2.3		1.4	1.4	1.4	1.4	1.3
[26]	*S. sikkimensis* (Ne8-2013) E-NEPAL	14.0	13.7	14.0	14.0	13.1	12.2	12.1	12.2	12.8	12.9	12.9	12.2	12.4	12.4	13.7	11.0	10.6	12.6	11.5	11.7	12.8	15.1	14.9	12.9	13.7		1.2	1.4	1.4	1.3
[27]	*S. sikkimensis* (JS140527) E-NEPAL	14.0	13.7	14.0	14.0	13.1	13.3	13.7	12.6	15.8	16.0	13.3	12.2	12.4	12.4	13.7	13.7	13.5	13.3	12.9	13.5	14.4	15.1	14.4	13.7	14.0	9.0		1.4	1.4	1.5
[28]	*S. sikimmensis* (KU243058) YADONG	14.6	14.0	14.6	14.2	14.6	13.7	11.9	13.8	12.1	12.1	14.2	13.8	13.7	13.8	14.2	13.5	13.5	14.6	12.6	12.4	12.1	13.8	13.3	11.3	12.1	13.1	12.4		0.8	1.4
[29]	*S. sikimmensis* (KU243059) YADONG	14.6	14.0	14.6	14.4	13.7	12.8	12.6	13.5	12.9	12.9	14.0	12.8	12.9	12.8	14.0	13.7	12.9	14.6	12.4	12.6	13.3	14.4	13.7	11.9	12.6	12.4	12.8	4.3		1.4
[30]	*S. spinosus* (KU243053) E-TIBET	12.8	12.6	12.8	12.6	12.2	11.2	12.1	11.7	10.1	10.3	12.6	11.5	11.7	11.3	12.6	10.4	9.4	10.4	11.7	11.0	9.9	14.2	13.3	11.9	11.9	11.7	14.0	11.9	12.4	
[31]	*S. wuguanfui* (KU243060) E-TIBET	14.9	14.6	14.9	14.7	16.2	15.1	14.7	16.2	16.7	16.9	16.2	15.8	16.0	16.0	14.4	15.8	15.5	14.6	15.8	15.6	14.6	13.8	14.4	13.1	13.5	14.2	15.5	13.5	15.3	14.7

1. Che, J. et al. Universal COI primers for DNA barcoding amphibians. Molecular Ecology Resources **12**, 247–58 (2012).
2. Jiang, K. et al. A new species of the genus *Scutiger* (Anura: Megophryidae) from Medog of southeastern Tibet, China. Zoological Research **37**:21–30 (2016).
3. Meng, H., Li, X., & Qiao, P. Population structure, historical biogeography and demographic history of the alpine toad *Scutiger ningshanensis* in the Tsinling Mountains of Central China. PloS One 9, e100729 (2014).
4. Su, L.-N., Meng, H.-Z., Li, X.-C. Genetic diversity and historical demography of the narrow-range endemic Alpine toad, *Scutiger liupanensis*, in the Liupan Mountains of central China. Genetics and Molecular Research **14**, 4865–78 (2015).

Table S5. Taxa, sample ID or voucher numbers, sample localities and GenBank accession numbers. The lower part of the table corresponds to the sequences used for the taxonomical evaluation of *Scutiger occidentalis* based on mitochondrial *co1* sequence data (for details see text). The "Clade; lineage/locality" label corresponds to Fig. 2 and 3, and S1-S5: GH=Greater Himalaya, THS=Tibet & Hengduan Shan clade, TMS=Tsinling Mountains & Sichuan Basin; C-Tibet=Central Tibet; E-Tibet=East Tibet; EN=East Nepal; SX=Shaanxi; WN=West Nepal; Dhaulg. N/S =Dhaulagiri Himal North/South, DuK. E/W=Dudh Koshi River East/West.; Nings.=Ningshan; NHAR=Ningxia Hui Autonomous Region, TAR=Tibet Autonomous Region; CIB= Chengdu Institute of Biology, NME=Natural History Museum of Erfurt, CAS=Chinese Academy of Science. Coordinates are given in degree, minutes.

Taxon	*Clade; lineage/locality*	Sample ID	origin	Sample site	Lat	Long	Alt	mtDNA (*16s*/*co1*/*cytb*/*nd4*)	nDNA (*bfib7*/*ccnb2intron3*/*rag1*)
S. cf. *boulengeri*	THS; SICHUAN	KQ2_2014	this study	China, Sichuan, Garzê	30°22'	101°40'	4062	KY310750/KY310860/KY310912/KY310972	KY310809/–/KY311026
S. cf. *boulengeri*	THS; SICHUAN	KQ3_2014	this study	China, Sichuan, Garzê	30°22'	101°40'	4062	KY310751/KY310861/KY310913/KY310973	KY310810/KY352189/KY311027
S. cf. *boulengeri*	THS; SICHUAN	KQ4_2014	this study	China, Sichuan, Garzê	30°22'	101°40'	4062	KY310752/KY310862/KY310914/KY310974	KY310811/KY352190/KY311028
S. cf. *boulengeri*	THS; SICHUAN	KQ5_2014	this study	China, Sichuan, Garzê	30°22'	101°40'	4062	KY310753/KY310863/KY310915/KY310975	KY310812/–/KY311029
S. cf. *boulengeri*	THS; SICHUAN	KQ6_2014	this study	China, Sichuan, Garzê	30°22'	101°40'	4062	KY310754/KY310864/KY310916/KY310976	KY310813/KY352191/KY311030
S. cf. *boulengeri*	THS; SICHUAN	KQ7_2014	this study	China, Sichuan, Garzê	30°22'	101°40'	4062	KY310755/KY310865/KY310917/KY310977	KY310814/KY352192/KY311031
S. cf. *boulengeri*	THS; SICHUAN	KQ8_2014	this study	China, Sichuan, Garzê	30°22'	101°40'	4062	KY310756/KY310866/KY310918/KY310978	–/KY352193/KY311032
S. cf. *boulengeri*	THS; SICHUAN	KQ12_2014	this study	China, Sichuan, Garzê	30°22'	101°40'	4062	KY310757/KY310867/KY310919/KY310979	KY310815/–/KY311033
S. cf. *boulengeri*	THS; SICHUAN	KQ14_2014	this study	China, Sichuan, Garzê	30°22'	101°40'	4062	KY310758/KY310868/KY310920/KY310980	KY310816/KY352194/KY311034
S. cf. *boulengeri*	THS; SICHUAN	KQ16_2014	this study	China, Sichuan, Garzê	30°22'	101°40'	4062	KY310759/KY310869/KY310921/KY310981	KY310817/–/KY311035
S. boulengeri	THS; C-TIBET	A1-AL	this study	China, TAR, Tageija	29°36'	85°44'	5067	KY310760/KY310870/KY310922/KY310982	KY310818/KY352195/KY311036
S. boulengeri	THS; C-TIBET	A2-AL	this study	China, TAR, Tageija	29°36'	85°44'	5067	KY310761/KY310871/KY310923/KY310983	KY310819/–/KY311037
S. boulengeri	THS; C-TIBET	A3-AL	this study	China, TAR, Damxung	30°09'	90°38'	4887	KY310762/KY310872/KY310924/KY310984	KY310820/–/KY311038
S. boulengeri	THS; C-TIBET	A4-AL	this study	China, TAR, Damxung	30°09'	90°38'	4887	KY310763/KY310873/KY310925/KY310985	*same as A3-AL*
S. boulengeri	THS; C-TIBET	A5-AL	this study	China, TAR, Damxung	30°09'	90°38'	4887	KY310764/KY310874/KY310926/KY310986	KY310821/KY352196/KY311039
S. boulengeri	THS; C-TIBET	JS1507_C1	this study	China, TAR, Lhasa	30°19'	91°31'	4400	KY310765/KY310875/KY310927/KY310987	KY310822/–/KY311040
S. boulengeri	THS; C-TIBET	JS1507_C2	this study	China, TAR, Lhasa	30°19'	91°31'	4400	KY310766/KY310876/KY310928/KY310988	KY310823/–/KY311041
S. boulengeri	THS; E-TIBET	CAS_XM1091	this study	China, TAR, Nyingchi	29°40'	94°42'	3850	KY310767/–/KY310929/KY310989	–/–/–
S. boulengeri	THS; E-TIBET	CAS_XM1095	this study	China, TAR, Nyingchi	29°40'	94°42'	3850	KY310768/KY310877/–/KY310990	–/–/–
S. boulengeri	THS; GANSU	jone1	NCBI	China, Gansu, Jone County	34°32'	103°29'		–/KJ082073/KJ082065/–	–/–/–
S. chintingensis	TMS; SICHUAN	JF174; ROM40460	NCBI	China, Sichuan	29°39'	102°57'		–/–/–/–	FJ945585/FJ945677/EF397301
S. chintingensis	TMS; SICHUAN	LC141	this study	China, Sichuan, Tianquan	30°14'	102°30'	2500	KY310769/KY310878/KY310930/KY310991	
S. chintingensis	TMS; SICHUAN	LC141_clone1_6	this study	China, Sichuan, Tianquan	30°14'	102°30'	2500		KY310824/KY352197/KY311042
S. chintingensis	TMS; SICHUAN	LC141_clone1_9	this study	China, Sichuan, Tianquan	30°14'	102°30'	2500		KY310825/KY352198/KY311043
S. glandulatus	THS; SICHUAN	SC1_2014	this study	China, Sichuan, Garzê	31°40'	099°43'	3457	KY310770/KY310879/KY310931/KY310992	KY310826/KY352199/KY311044
S. glandulatus	THS; SICHUAN	SC2_2014	this study	China, Sichuan, Garzê	29°47'	100°25'	3713	KY310771/KY310880/KY310932/KY310993	KY310827/KY352200/KY311045
S. glandulatus	THS; SICHUAN	KQ61_2014	this study	China, Sichuan, Garzê	29°47'	100°25'	3713	KY310772/KY310881/KY310933/KY310994	KY310828/KY352201/KY311046
S. glandulatus	THS; SICHUAN	SH150516	this study	China, Sichuan, Garzê	30°14'	101°30'	3526	*same as SC1_2014*	KY310829/–/KY311047
S. glandulatus	THS; SICHUAN	SH150531	this study	China, Sichuan, Kangding	30°02'	101°32'	3474	KY310773/KY310882/KY310934/KY310995	KY310830//KY311048
S. glandulatus	THS; SICHUAN	SH150557	this study	China, Sichuan, Garzê	29°47'	100°25'	3713	KY310774/KY310883/KY310935/KY310996	KY310831/–/KY311049

S. glandulatus	THS; SICHUAN	CIB_XM1188	NCBI	China, Sichuan, Xiangcheng	29°09'	99°56'		EF397274/–/FJ945466/–	–/–/–
S. cf. *mammatus*	THS; YUNNAN	Yako01	NCBI	China, Yunnan				EU180890/–/EU180932/EU180974	–/–/–
S. mammatus	THS; SICHUAN	XM972	NCBI	China, Sichuan	30°01'	101°28'		–/–/–/–	FJ945581/FJ945675/EF397300
S. mammatus	THS; SICHUAN	LC045	this study	China, Sichuan, Baiyu	31°01'	99°15'	3494	KY310775/KY310884/KY310964/KY310997	KY310832/KY352202/KY311050
S. nepalensis	GH; WN (Kanjiroba)	NME_A2014/13	this study	Nepal, Jumla	29°21'	82°09'	3285	KY310776/KY310885/KY310936/–	KY310833/–/KY311051
S. nepalensis	GH; WN (Saipal)	NME_A2018/13	this study	Nepal, Chainpur	29°22'	81°08'	3000	KY310777/KY310886/KY310937/KY310998	KY310834/KY352203/KY311052
S. nepalensis	GH; WN (Dhaulg. N)	A8-2012	this study	Nepal	28°42'	82°55'	3340	KY310778/KY310887/KY310938/KY310999	KY310835/KY352204/KY311053
S. nepalensis	GH; WN (Dhaulg. N)	A9-2012	this study	Nepal	28°36'	83°01'	3250	KY310779/KY310888/KY310939/KY311000	KY310836/KY352205/KY311054
S. nepalensis	GH; WN (Dhaulg. N)	A10-2012	this study	Nepal	28°36'	83°01'	3250	KY310780/KY310889/KY310940/KY311001	KY310837/–/KY311055
S. nepalensis	GH; WN (Dhaulg. N	A11-2012	this study	Nepal	28°36'	83°01'	3250	KY310781/KY310890/KY310941/KY311002	*same as A8-2012*
S. nepalensis	GH; WN (Dhaulg. N)	A12-2012	this study	Nepal	28°36'	83°01'	3250	KY310782/KY310891/KY310942/KY311003	KY310838/KY352207/KY311056
S. nepalensis	GH; WN (Dhaulg. N)	A13-2012	this study	Nepal	28°36'	83°01'	3250	KY310783/KY310892/KY310943/KY311004	KY310839/KY352208/KY311057
S. nepalensis	GH; WN (Dhaulg. S)	A14-2012	this study	Nepal	28°30'	83°02'	3073	KY310784/KY310893/KY310944/KY311005	KY310840/KY352209/KY311058
S. nepalensis	GH; WN (Dhaulg. N)	A15-2012	this study	Nepal	28°30'	83°07'	2993	KY310785/KY310894/KY310945/KY311006	KY310841/KY352210/KY311059
S. nepalensis	GH; WN (Dhaulg. N)	A16-2012	this study	Nepal	28°30'	83°07'	2993	KY310786/KY310895/KY310946/KY311007	–/–/–
S. nepalensis	GH; WN (Saipal)	MS_Scut2	this study	Nepal, Juphal	29°01'	82°47'	4450	KY310787/KY310896/KY310947/KY311008	*same as A2014-13*
S. nepalensis	GH; WN (Saipal)	MS_Scut5	this study	Nepal, Dhauli Lake	29°21'	82°23'	4449	–/–/–/–	KY310842/KY352211/KY311060
S. nepalensis	GH; WN (Saipal)	MS_Scut6	this study	Nepal, Dhauli Lake	29°21'	82°23'	4450	KY310788/KY310897/KY310948/–	KY310843/–/KY311061
S. nepalensis	GH; WN (Saipal)	MS_Scut7	this study	Nepal, Dhauli Lake	29°21'	82°23'	4450	KY310789/–/KY310949/-	KY310844/–/KY311062
S. nepalensis	GH; WN (Saipal)	MS_Scut8	this study	Nepal, Dhauli Lake	29°21'	82°23'	4450	KY310790/–/KY310950/-	KY310845/KY352212/KY311063
S. nepalensis	GH; WN (Saipal)	MS_Scut9	this study	Nepal, Dhauli Lake	29°21'	82°23'	4450	KY310791/KY310898/KY310951/KY311009	–/KY352213/KY311064
S. nepalensis	GH; WN (Saipal)	MS_Scut10	this study	Nepal, Bumra	29°23'	82°08'	2700	KY310792/–/KY310952/–	*same as A2014-13*
S. occidentalis	PAKISTAN	MS_PK1	this study	Pakistan, Deosai Plains	34°59'	75°15'	4100	KY310793/–/KY310953/KY311010	KY310846/KY352214/KY311065
S. occidentalis	PAKISTAN	MS_PK2	this study	Pakistan, Deosai Plains	34°59'	75°15'	4100	KY310794/KY310899/KY310954/KY311011	*same as MS_PK1*
S. occidentalis	PAKISTAN	MS_PK4	this study	Pakistan, Deosai Plains	34°59'	75°15'	4100	*same as MS_PK2*	KY310847/KY352216/KY311066
S. occidentalis	PAKISTAN	MS_PK5	this study	Pakistan, Deosai Plains	34°59'	75°15'	4100	KY310795/KY310900/KY310956/KY311012	*same as MS_PK1*
S. occidentalis	PAKISTAN	MS_PK6	this study	Pakistan, Deosai Plateau	35°2'	75°26'	4100	KY310796/KY310901/KY310957/KY311013	*same as MS_PK4*
S. sikkimensis	GH; EN (Arun)	JS140523	this study	Nepal, Kongma Danda	27°38'	87°13'	3000	KY310797/–/KY310958/KY311014	KY310848/–/KY311067
S. sikkimensis	GH; EN (Arun)	JS140524	this study	Nepal, Kongma Danda	27°38'	87°13'	3000	KY310798/KY310902/KY310959/KY311015	KY310849/–/KY311068
S. sikkimensis	GH; EN (Arun)	JS140525	this study	Nepal, Kongma Danda	27°38'	87°13'	3000	KY310799/KY310903/KY310960/KY311016	KY310850/KY352219/KY311069
S. sikkimensis	GH; EN (Arun)	JS140526	this study	Nepal, Kongma Danda	27°38'	87°13'	3000	KY310800/–/KY310961/KY311017	KY310851/KY352220/KY311070
S. sikkimensis	GH; EN (Arun)	JS140527	this study	Nepal, Kongma Danda	27°38'	87°13'	3000	*same as JS140525*	KY310852/–/KY311071
S. sikkimensis	GH; EN (Arun)	JS140528	this study	Nepal, Kongma Danda	27°38'	87°13'	3000	KY310801/KY310904/KY310963/KY311018	KY310853/KY352221/KY311072
S. sikkimensis	GH; EN (DuK. W)	Ne5-2013	this study	Nepal, Surkie La	27°34'	86°48'	2900	KY310802/KY310905/KY310965/KY311019	KY310854/KY352222/KY311073
S. sikkimensis	GH; EN (DuK. W)	Ne6-2013	this study	Nepal, Surkie La	27°34'	86°48'	3000	KY310803/KY310906/KY310966/KY311020	KY310855/KY352223/KY311074
S. sikkimensis	GH; EN (DuK. W)	Ne7-2013	this study	Nepal, Surkie La	27°34'	86°48'	3000	KY310804/KY310907/KY310967/KY311021	KY310856/KY352224/KY311075
S. sikkimensis	GH; EN (DuK. W)	Ne8-2013	this study	Nepal, Surkie La	27°35'	86°50'	3550	KY310805/KY310908/KY310968/KY311022	KY310857/KY352225/KY311076
S. sikkimensis	GH; EN (DuK. E)	Ne11-2013	this study	Nepal, Taksindo	27°39'	86°36'	3900	KY310806/KY310909/KY310969/KY311023	KY310858/KY352226/KY311077
S. sikkimensis	GH; EN (DuK. E)	Ne14-2013	this study	Nepal, Lamajura La	27°34'	86°30'	3100	KY310807/KY310910/KY310970/KY311024	KY310859/–/KY311078
S. sikkimensis	GH; EN (DuK. E)	Ne15-2013	this study	Nepal, Lamajura La	27°34'	86°30'	3400	KY310808/KY310911/KY310971/KY311025	*same as Ne14-2013*
S. liupanensis	THS; LIUPAN MTS	FWR11	NCBI	China, Liupan Mountains	35°30'	106°15'		–/KC140483/JX533792/–	–/–/–
S. liupanensis	THS; LIUPAN MTS	CPG8	NCBI	China, Liupan Mountains	35°22'	106°18'		–/KC140479/JX533802/–	–/–/–
S. liupanensis	THS; LIUPAN MTS	CPG9	NCBI	China, Liupan Mountains	35°22'	106°18'		–/KC140491/JX533804/–	–/–/–

S. liupanensis	THS; LIUPAN MTS	SSR14	NCBI	China, Liupan Mountains	35°21'	105°19'		–/KC140478/JX533796/–	–/–/–
S. liupanensis	THS; LIUPAN MTS	SSR23	NCBI	China, Liupan Mountains	35°21'	106°19'		–/KC140508/JX533795/–	–/–/–
S. liupanensis	THS; LIUPAN MTS	DDR10	NCBI	China, Liupan Mountains	35°20'	106°20'		–/KC140474/JX533805/–	–/–/–
S. liupanensis	THS; LIUPAN MTS	MXF11	NCBI	China, Liupan Mountains	35°10'	106°29'		–/KC140475/JX533806/–	–/–/–
S. liupanensis	THS; LIUPAN MTS	MXF1	NCBI	China, Liupan Mountains	35°10'	106°29'		–/KC140513/JX533803/–	–/–/–
S. ningshanensis	TMS; SX (Henan)	bys1	NCBI	China, Henan, Songxian Co.	33°39'	111°49'	1675	–/KF757397/KF757340/–	–/–/–
S. ningshanensis	TMS; SX (Taibai)	hby1	NCBI	China, Shaanxi, Taibai Co	33°52'	107°31'	1652	–/KF757402/KF757366/–	–/–/–
S. ningshanensis	TMS; SX (Taibai)	hby2	NCBI	China, Shaanxi, Taibai Co	33°52'	107°31'	1652	–/KC140559/KC140561/–	–/–/–
S. ningshanensis	TMS; SX (Taibai)	hby3	NCBI	China, Shaanxi, Taibai Co	33°52'	107°31'	1652	–/KC140560/KC140562/–	–/–/–
S. ningshanensis	TMS; SX (Taibai)	hby7	NCBI	China, Shaanxi, Taibai Co	33°52'	107°31'	1652	–/KF757412/KF757371/–	–/–/–
S. ningshanensis	TMS; SX (Taibai)	hby13	NCBI	China, Shaanxi, Taibai Co	33°52'	107°31'	1652	–/KF757405/KF757342/–	–/–/–
S. ningshanensis	TMS; SX (Taibai)	hby19	NCBI	China, Shaanxi, Taibai Co	33°52'	107°31'	1652	–/KF757408/KF757344/–	–/–/–
S. ningshanensis	TMS; SX (Taibai)	lfy2	NCBI	China, Shaanxi, Foping Co	33°40'	107°51'	2047	–/KF757420/KF757377/–	–/–/–
S. ningshanensis	TMS; SX (Taibai)	lfy5	NCBI	China, Shaanxi, Foping Co	33°40'	107°51'	2047	–/KF757422/KF757378/–	–/–/–
S. ningshanensis	TMS; SX (Taibai)	lfy17	NCBI	China, Shaanxi, Foping Co	33°40'	107°51'	2047	–/KF757419/KF757376/–	–/–/–
S. ningshanensis	TMS; SX (Nings.)	nsc1	NCBI	China, Shaanxi, Ningshan Co	33°28'	108°31'	2000	–/KF757427/KF757355/–	–/–/–
S. ningshanensis	TMS; SX (Nings.)	nsc2	NCBI	China, Shaanxi, Ningshan Co	33°28'	108°31'	2000	–/KF757433/KF757386/–	–/–/–
S. ningshanensis	TMS; SX (Nings.)	nsc10	NCBI	China, Shaanxi, Ningshan Co	33°28'	108°31'	2000	–/KF757428/KF757356/–	–/–/–
S. ningshanensis	TMS; SX (Henan)	ljs18	NCBI	China, Henan, Luanchuan Co.	33°43'	111°37'	1590	–/KF757425/KF757353/–	–/–/–
S. ningshanensis	TMS; SX (Henan)	srs15	NCBI	China, Henan, Lushan Co.	33°43'	112°15'	1642	–/KF757437/KF757362/–	–/–/–
S. sp.	THS; YUNNAN	CAS228188	NCBI	China, Yunnan	27°50'	98°50'		EU180889/–/EU180931/EU180973	–/–/–
S. tuberculatus	THS; SICHUAN	CIBXM988	NCBI	China, Sichuan	28°31'	102°30'		EF397278/–/FJ945493/–	–/–/–
Alytes obstetricans			NCBI					mt-genome: NC_006688	–/–/–
Bombina bombina			NCBI					mt-genome: JX893172	–/–/–
Discoglossus galganoi			NCBI					mt-genome: AY585339	–/–/–
Leptobrachium boringii		SCUM120630	NCBI	China, Sichuan				mt-genome: NC_024427	–/–/–
Oreolalax chuanbeiensis		CIBXM074/ DQR_Pingwu_001J	NCBI	China, Sichuan, Mao Xian Co/ Pingwu Co.	31°45'	104°07'		EF397266/–/EU180929/EU180971	–/–/–
Oreolalax omeimontis		CIBXM439/ KIZOO96002	NCBI	China, Sichuan, Emei Mt.	29°33'	103°22'		EF397264/–/EU180928/EU180970	–/–/–
Megophrys sp.			NCBI					mt-genome: KT601071	–/–/–
Pelobates cultripes			NCBI	Spain, Madrid				mt-genome: AJ871086	–/–/–
Pelodytes punctatus			NCBI					mt-genome: NC_020000	–/–/–
Pipa pipa			NCBI					mt-genome: GQ244477	–/–/–
Rhinophrynus dorsalis			NCBI					mt-genome: JX564892	–/–/–
Scaphiopus			NCBI					AB612078/AB612075/JX564894/JX564894	–/–/–
								co1	
S. boulengeri	–; C-TIBET	KIZ08195	NCBI	China, TAR				JN700837[#]	
S. boulengeri	–; E-TIBET	KIZ06712	NCBI	China, TAR, Bomi				KU243064*	
S. boulengeri	–; GANSU	Jone1	NCBI	China, Gansu				KJ082073[‡]	
S. gongshanensis	–; YUNNAN	CIB20070717001	NCBI	China, Yunnan, Gongshan	27°42'	98°30'		KU243062[#]	
S. gongshanensis	–; YUNNAN	CIB20070717002	NCBI	China, Yunnan, Gongshan	27°42'	98°30'		KU243063[#]	
S. jiulongensis	–; SICHUAN	KIZ045055	NCBI	China, Sichuan, Ganzi				KU243066[#]	
S. liupanensis	–; LIUPAN MTS	FWR11	NCBI	China, NHAR				KC140483[†]	

S. liupanensis	–; LIUPAN MTS	CPG8	NCBI	China, NHAR	KC140479[†]
S. liupanensis	–; LIUPAN MTS	DDR10	NCBI	China, NHAR	KC140474[†]
S. nyingchiensis	–; E-TIBET	KIZ017459	NCBI	China, TAR, Nyingchi, Medog	KU243056*
S. ningshanensis	–; SX	hby19	NCBI	China, Shaanxi, Taibai Co	KF757408[‡]
S. ningshanensis	–; SX	lfy17	NCBI	China, Shaanxi, Foping Co	KF757419[‡]
S. ningshanensis	–; SX	nsc1	NCBI	China, Shaanxi, Ningshan Co	KF757427[‡]
S. ningshanensis	–; SX	srs15	NCBI	China, Henan, Lushan Co	KF757437[‡]
S. sikimmensis	–; YADONG	KIZ011127	NCBI	China, TAR, Yadong	KU243058
S. sikimmensis	–; YADONG	KIZ07371	NCBI	China, TAR, Yadong	KU243059
S. spinosus	–; E-TIBET	KIZ011114	NCBI	China, TAR, Nyingchi, Medog	KU243053
S. wuguanfui	–; E-TIBET	KIZ011101	NCBI	China, TAR, Nyingchi, Medog	KU243060

[#]Che, J. et al. Universal COI primers for DNA barcoding amphibians. Molecular Ecology Resources **12**, 247–58 (2012).

*Jiang, K. et al. A new species of the genus *Scutiger* (Anura: Megophryidae) from Medog of southeastern Tibet, China. Zool Res. **37**, 21–30 (2016).

[‡]Meng, H., Li, X. & Qiao, P. Population structure, historical biogeography and demographic history of the alpine toad *Scutiger ningshanensis* in the Tsinling Mountains of Central China. PLoS One **9**, e100729 (2014).

[†]Su, L.-N., Meng, H.-Z. & Li, X.-C. Genetic diversity and historical demography of the narrow-range endemic Alpine toad, *Scutiger liupanensis*, in the Liupan Mountains of central China. Genetics and Molecular Research **14**, 4865–78 (2015).

Table S6. Primer and annealing temperature used for DNA amplification (amp) and sequencing (seq).

gene	*Primer*		*Direction*	*Sequence 5' → 3'*	*annealing*	*Reference*
16S ribosomal RNA (*16S*)	L02510	amp + seq	forward	CGCCTGTTTAYCAAAAACAT	52°, 120 sec	[1]
	H03063	amp + seq	reverse	CTCCGGTTTGAACTCAGATC	52°, 120 sec	[2]
cytochrome oxidase subunit 1 (*co1*)	Chmf4	amp + seq	forward	TYTCWACWAAYCAYAAAGAYATCGG	50°, 60 sec	[3]
	Chmr4	amp + seq	reverse	ACYTCRGGRTGRCCRAARAATCA	50°, 60 sec	[3]
cytochrome b (*cytb*)	SCU_L14841	amp + seq	forward	CTTCCATCCAACATCTCAGCATGATGAAA	50°, 90 sec	[4]
	SCU5	amp + seq	reverse	ACAAGACCAATGCTTTAGTTAAGCTAC	50°, 90 sec	[5]
dehydrogenase subunit 4 (*nd4*)	nd4_1-F	amp + seq	forward	CTATTCTTCTAAAATTAGGGGGATATGG	50°, 60 sec	this study
	nd4_1-R	amp + seq	reverse	GGATTACTATTTTCCTTTAAAAGCCG	50°, 60 sec	this study
beta-Fibrinogen intron 7 (*bfib7*)	BFX-F1	amp + seq	forward	ACCCAAACTACAGTATGATGCC	57°, 60 sec	[6]
	BFX-R1	amp + seq	reverse	TTCCTCACCACCTTACTTCCT	57°, 60 sec	[6]
cyclin B2 gene intron 3 (*ccnb2*)	ccnb_SF	amp + seq	forward	GAATGTGGACTCTAATGGAGAAG	57°, 60 sec	[6]
	ccnb_SR1	amp + seq	reverse	TGGAGGAATAGCCGCTTTATGC	57°, 60 sec	[6]
recombination activating protein 1 gene (*rag1*)	Amp-RAG1_F	amp	forward	AGCTGCAGYCARTACCAYAARATGTA	56°, 90 sec	[7]
	Amp-RAG1_R	amp	reverse	GCAAAGTTTCCGTTCATTCTCAT	56°, 90 sec	[8]
	rag1_Scut_Intern_F	seq	forward	TGTCAAAGCCACAACTGGAAGG		this study
	rag1_Scut_Intern_F	seq	forward	TCATTTTCTTCCTGAGGTGTTTGTC		this study

1. Palumbi, S. R. Nucleic Acids II: the polymerase chain reaction in *Molecular Systematics* (eds Hillis, D.M., Moritz, C. & Mable, B. K.) 205–47 (Sinauer Associates, 1996).
2. Rassmann, K. Evolutionary age of the Galapagos iguanas predates the age of the present Galapagos Islands. Molecular Phylogenetics and Evolution **7**, 158–72 (1997).
3. Che, J. et al. Universal COI primers for DNA barcoding amphibians. Molecular Ecology Resources **12**, 247–58 (2012).
4. Kocher, T. D. et al. Dynamics of mitochondrial DNA evolution in animals: amplification and sequencing with conserved primers. Proceedings of the National Academy of Sciences USA **86**, 6196–200 (1989).
5. Li, R. et al. Rivers as barriers for high elevation amphibians: a phylogeographic analysis of the alpine stream frog of the Hengduan. Journal of Zoology **277**, 309–16 (2009).
6. Chen, W., Bi, K. & Fu, J. Frequent mitochondrial gene introgression among high elevation Tibetan megophryid frogs revealed by conflicting genegenealogies. Molecular Ecology **18**, 2856–76 (2009).
7. Mauro, D. S., Gower, D. J., Oommen, O. V., Wilkinson, M. & Zardoya, R. Phylogeny of caecilian amphibians (Gymnophiona) based on complete mitochondrial genomes and nuclear RAG1. Molecular Phylogenetics and Evolution **33**, 413–27 (2004).
8. Fu, J., Weadick, C. J. & Bi, K. A phylogeny of the high-elevation Tibetan megophryid frogs and evidence for the multiple origins of reversed sexual size dimorphism. Journal of Zoology **273**, 315–25 (2007).
